# Autographa californica multiple nucleopolyhedrovirus Ac51 interacts with Ac66 and facilitates its nuclear localization to promote the nuclear egress of nucleocapsids

**DOI:** 10.1128/jvi.01969-24

**Published:** 2025-05-28

**Authors:** Jianxiang Qiu, Guo-Sheng Zhu, Jiaxin Liu, Longkuan Feng, Zefen Pang, Dandong Zhu, Kanghong Chen, Xiukui Yan, Ao Li, Chuming You, Zhixin Fang

**Affiliations:** 1 Biosafety Laboratory, The Affiliated Guangdong Second Provincial General Hospital of Jinan University 485285 , Guangzhou, Guangdong, China; 2 GuangDong Engineering Technology Research Center of Emergency Medicine, The Affiliated Guangdong Second Provincial General Hospital of Jinan University 485285 , Guangzhou, Guangdong, China; 3 School of Life Sciences and Medicine, Shandong University of Technology 91620, Zibo, Shandong, China; The University of Arizona, Tucson, Arizona, USA

**Keywords:** AcMNPV, Ac51, Ac66, nucleocapsid egress

## Abstract

Autographa californica multiple nucleopolyhedrovirus is a large DNA virus that replicates within host nuclei. The mechanism underlying the nuclear egress of viral nucleocapsids is not fully understood. We previously identified Ac51 as a key player in facilitating efficient nuclear egress of nucleocapsids for the release of budded viruses (BVs). This protein joins Ac66 and EXON0 as the third identified protein with a similar function. In this study, we evaluated the functional importance of conserved domains of Ac51 during virus replication and found that the coiled-coil (CC) motif of Ac51 was crucial for the protein’s accumulation and BV production. Our study further revealed a strong interaction and colocalization between Ac51 and Ac66. Deletion of *ac51* significantly impaired the nuclear import of Ac66, leading to its aggregation in the cytoplasm. Deletion of the CC motif also significantly reduced the nuclear localization of Ac66 and their colocalization, while deletion of the DnaJ domain of Ac51 seemed to impair the interaction between Ac51 and Ac66 *in vitro*. Molecular docking analysis provided a potential interaction mechanism between Ac51 and Ac66. Intriguingly, our study also identified two other viral nucleocapsid proteins, ME53 and Ac132, which interacted and colocalized with both Ac51 and Ac66. Molecular docking analysis suggested distinct binding regions of ME53 and Ac132 within the Ac51-Ac66 complex. In summary, these results demonstrated that Ac51 and Ac66 cooperate to facilitate the nuclear egress of nucleocapsids and uncovered a functional correlation between ME53/Ac132 and the Ac51-Ac66 complex in nucleocapsid assembly or transport.

**IMPORTANCE:**

The nuclear egress of AcMNPV nucleocapsids is a crucial step for producing high levels of budded viruses, but its underlying mechanism is not fully understood. Previous studies have highlighted the significant roles of three nucleocapsid proteins, including Ac51, Ac66, and EXON0, in facilitating efficient nuclear egress of nucleocapsids. Here, we demonstrated a strong interaction and colocalization between Ac51 and Ac66, but not between Ac51 and EXON0. Furthermore, Ac51 is required for efficient nuclear localization of Ac66, indicating that Ac51 exerts a role in Ac66 to promote nucleocapsid egress. Our study also identified two other nucleocapsid proteins, ME53 and Ac132, which interacted and colocalized with both Ac51 and Ac66, suggesting their functional correlation in nucleocapsid assembly or transport. These discoveries enhanced our understanding of the functional mechanism of Ac51 and nucleocapsid assembly and transport and provided insights for further investigation of the nucleocapsid transport process.

## INTRODUCTION

The antegrade transport of viral nucleocapsids represents the transportation of newly synthesized nucleocapsids from their assembly sites to the plasma membrane for budding. This process is an obligatory step of viral maturation and also a good target for the disruption of viral infection. The transportation relies on the cellular microtubule or microfilament system (1–4) and involves the coordination of various viral and host proteins (5–9).

Baculoviruses are large enveloped, circular, double-stranded DNA viruses with genome sizes ranging from approximately 80 to 180 kDa, which are specifically pathogenic to insects. The mechanism underlying the antegrade transport of its nucleocapsids to form mature viruses is largely unknown. A typical feature of the baculovirus infection cycle is the production of two physically different but genome-identical types of virions: budded viruses (BVs) and occlusion-derived viruses (ODVs). BVs mediate the spread of the virus between cells, while ODVs facilitate the transmission of the virus among insects. The most extensively studied baculovirus is Autographa californica multiple nucleopolyhedrovirus (AcMNPV), species *Alphabaculovirus aucalifornicae*, which is the archetype species of the genus *Alphabaculovirus*. The life cycle of AcMNPV within cells involves the following key processes: BVs enter cells through endocytosis and release nucleocapsids into the cytoplasm. The nucleocapsids are then transported through the microfilament system to the nuclear pore complex, pass through the nuclear pore, and enter the cell nucleus, where viral DNA is released (7, 10). In the nucleus, the virus induces the formation of virogenic stroma (VS) to initiate viral replication. Through DNA replication, gene transcription, and protein translation, new nucleocapsids are assembled within the VS. In the early phase of infection, newly formed nucleocapsids are transported from the VS to the nuclear membrane, cross the nuclear membrane and the cytoplasm, and finally reach the plasma membrane, where they bud off to acquire a membrane-derived envelope and form mature BVs (7, 11). In the late phase of infection, nucleocapsids remain in the nucleus. They are enveloped by microvesicles derived from the nuclear membrane to form ODVs. ODVs are eventually embedded in protein crystals to form occlusion bodies, which can remain biologically active and stably exist in the environment (11, 12).

To date, the anterograde transport of baculoviruses is not fully understood. Within the VS, nucleocapsids are assembled and subsequently transported to the ring zone with the assistance of nuclear F-actin and viral structural proteins, such as VP80 (13). The exact mechanism by which nucleocapsids traverse the nuclear membrane has not been definitively determined. However, extensive transmission electron microscopy data suggest that nucleocapsids bud through the nuclear membrane via membrane fusion (7, 14). Several viral nucleocapsid proteins and ODV envelope proteins have been identified as crucial for this process. These include Ac11, GP41, P48, Ac93, Ac75, Ac76, and Ac142 (15–22). While the absence of any of these proteins did not affect visible nucleocapsid assembly, it completely blocked nucleocapsid budding, BV production, and ODV formation. Furthermore, cellular proteins involved in membrane vesicle fusion, membrane remodeling, and scission, such as N-ethylmaleimide-sensitive factor (NSF) and the endosomal sorting complex required for transport III (ESCRT-III) complex, have been found to interact directly or indirectly with these viral structural proteins. Interfering with the function of the NSF or ESCRT-III complex has been shown to decrease the nuclear egress of nucleocapsids and BV production (14, 23, 24). This suggests that the viral proteins mentioned earlier may form an “egress complex” and could potentially recruit components of the ESCRT-III complex to a specific virus-egress domain on the nuclear membrane to facilitate the budding of nucleocapsids through the nuclear membrane.

Certain viral nucleocapsid proteins, such as EXON0, Ac66, and Ac51 (named according to the book *Baculovirus Molecular Biology* [11]), have been identified in previous studies to play a role in facilitating efficient nuclear egress of nucleocapsids through a distinct mechanism that is not yet fully understood. The absence of any of these proteins did not disrupt visible nucleocapsid assembly or block the nuclear egress of nucleocapsids and ODV formation (25–27). However, their deficiency led to a significant reduction in the number of nucleocapsids during nuclear egress and subsequent transport, resulting in a dramatic reduction in BV production by 1,000- to 10,000-fold, severely impeding viral spread. Among these three proteins, EXON0 was the most extensively studied. It has been found to be associated with microtubules, as evidenced by its interaction and colocalization with tubulin and kinesin. This suggests that EXON0 may play a role in mediating the transport of nucleocapsid along microtubules (28–30). A further study has demonstrated that EXON0 and Ac66 interact with each other and colocalize within the VS of infected cells (31). This study also proposes that the ubiquitination of nucleocapsids serves as one of the signals to facilitate their nuclear egress, with Ac66 being one of the proteins ubiquitinated on the nucleocapsids (31). To date, the precise mechanism by which Ac51 promotes the nuclear egress of nucleocapsids and its functional relationship with Ac66 and EXON0 remains unclear.

In our previous study, we investigated the role of Ac51 in the AcMNPV life cycle. Our findings revealed that Ac51 is a nucleocapsid protein and is distributed in both the nucleus and cytoplasm of infected cells. By generating a recombinant virus with the deletion of the *ac51* gene, we found that the absence of Ac51 did not affect viral DNA replication, viral gene expression, and virus morphogenesis. However, the deletion significantly impeded the nuclear egress of nucleocapsids and subsequent transportation to form BVs, indicating that Ac51 is involved in the biological processes associated with the anterograde transport of nucleocapsids (27). Furthermore, the deletion of *ac51* also resulted in a loss of virulence *in vivo*. Bioinformatic analysis showed that Ac51 contains three conserved domains: an N-terminal DnaJ domain, a middle RNA recognition domain (RRM), and a C-terminal coiled-coil (CC) motif (27, 32). However, the functional significance of these domains remains unknown.

In the present study, we aimed to further investigate the functional mechanism of Ac51 in the process of nuclear egress of nucleocapsids to release progeny viruses. Our results showed that the CC motif played a crucial role in the accumulation of Ac51 in infected cells and is most important for virus multiplication compared with the DnaJ domain and RRM. Our study further revealed that Ac51 interacted with Ac66 instead of EXON0 and exhibited a high degree of colocalization with Ac66 within infected cells. Deletion of Ac51 significantly impaired the nuclear import of Ac66, leading to its aggregation in the cytoplasm. We also revealed the interaction mechanism between Ac51 and Ac66. Furthermore, our study uncovered two other nucleocapsid proteins, ME53 and Ac132, which interacted and colocalized with both Ac51 and Ac66 during virus infection, suggesting their functional correlation in the process of nucleocapsid assembly or transport.

## RESULTS

### Effect of Ac51 domain deletion on virus replication

**Fig 1:**
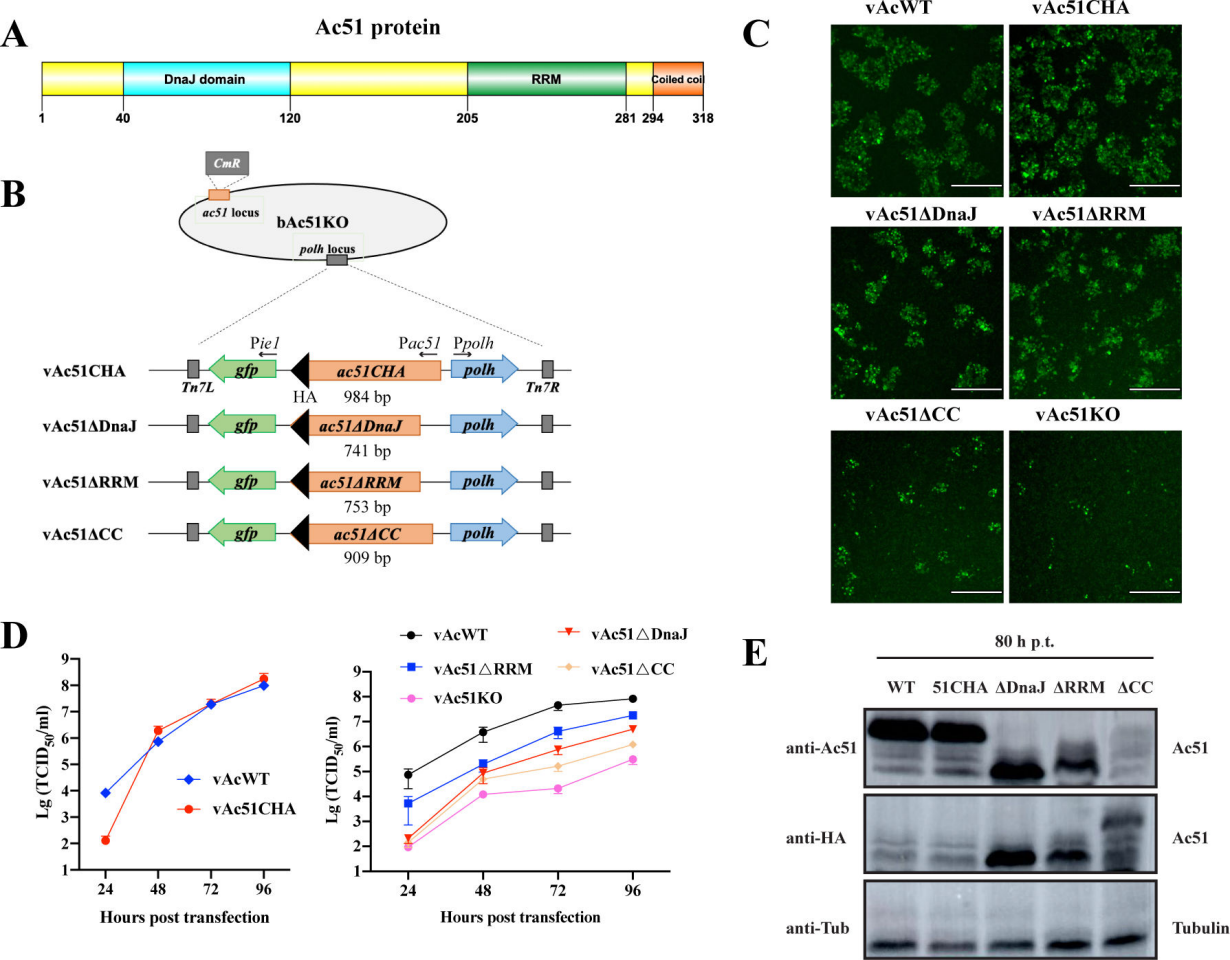
Functional analysis of Ac51 domains. (**A**) A schematic diagram illustrating the conserved domains of Ac51. The picture was generated using DOG 2.0 (33). The amino acid regions corresponding to each domain in the Ac51 protein were indicated. (**B**) The construction strategy employed for generating domain-deleted recombinant viruses. (**C**) Replication of recombinant viruses. Sf9 cells were transfected with 0.2 µg of bacmid DNA of indicated recombinant viruses. The spread of viruses, indicated by GFP expression, was analyzed using plaque assays and observed at 120 h p.t. using a fluorescence microscope. The bar represents 330 µm. (**D**) Virus growth curves of recombinant viruses. Sf9 cells were transfected with 1 µg of bacmid DNA of indicated recombinant viruses, and cell supernatant was collected at 24, 48, 72, and 96 h p.t. Virus titer was determined by the TCID_50_ endpoint dilution assay. The data represent three independent biological replicates. (**E**) Expression of truncated Ac51 in infected cells. Sf9 cells transfected with bacmid DNA of indicated recombinant viruses were collected at 72 h p.t. and subjected to western blot analysis.

Bioinformatic analysis revealed that Ac51 possesses an N-terminal DnaJ domain, a middle RRM, and a C-terminal CC motif (Fig. 1A). We first examined the functional roles of these domains in virus replication. Recombinant viruses with deletions in each domain were generated, namely vAc51ΔDnaJ, vAc51ΔRRM, and vAc51ΔCC (Fig. 1B). An HA tag was fused to the C-terminal end of the truncated Ac51 to facilitate detection. An HA-tagged full-length Ac51 was constructed as a control, and vAc51CHA is referred to as the Ac51 repair virus (Fig. 1B). Sf9 cells were transfected with bacmid DNA of the recombinant viruses, and virus spread was assessed by plaque assays. As shown in Fig. 1C, the deletion of Ac51 severely hindered virus spread, consistent with the findings in our previous study (27). Furthermore, deletion of any of the three domains also impaired virus spread (Fig. 1C). Analysis of the virus growth curves revealed comparable replication ability between vAc51CHA and vAcWT, indicating that vAc51CHA can restore virus replication. The difference in viral titers at 24 hours post-transfection (h p.t.) likely arose from variations in transfection efficiency, as fluorescence microscopy indicated that the transfection efficiency of vAc51CHA was lower than that of vAcWT. Additionally, a significant reduction in virus production was observed following the deletion of any of the three domains. At 96 h p.t., deletion of *ac51* resulted in an approximately 150-fold decrease in virus production. Deletion of the DnaJ domain or RRM led to approximately 15- and 5-fold reductions, respectively. Deletion of the CC motif resulted in a 70-fold decrease in virus production (Fig. 1D). Interestingly, when the cell culture medium was supplemented with 100 µg/mL penicillin and 30 µg/mL streptomycin (the culture condition in our previous study [27]), the reduction in virus production upon domain deletion was more pronounced. In this medium, at 96 h p.t., deletion of *ac51* resulted in an approximately 1,600-fold decrease in virus production, while deletion of the DnaJ domain and the RRM led to approximately 140- and 130-fold reductions, respectively. Deletion of the CC motif resulted in a 1,500-fold decrease in virus production (Fig. S1). These results were consistent with the titer results in our previous study (27). Western blot analysis with an anti-Ac51 antibody showed that full-length Ac51, Ac51CHA, Ac51ΔDnaJ, and Ac51ΔRRM were detectable in infected cells, whereas Ac51ΔCC showed extremely low abundance in the cells (Fig. 1E). This reduced expression of Ac51ΔCC in infected cells may be responsible for the severely impaired virus titer observed. Additionally, although Ac51ΔDnaJ, Ac51ΔRRM, and Ac51ΔCC were detectable with an anti-HA antibody, full-length Ac51CHA was not. Taken together, these results indicated that all conserved domains of Ac51 are necessary for efficient BV production and its overall function.

### Deletion of the CC motif reduced the abundance of Ac51 in infected cells and on virions

**Fig 2:**
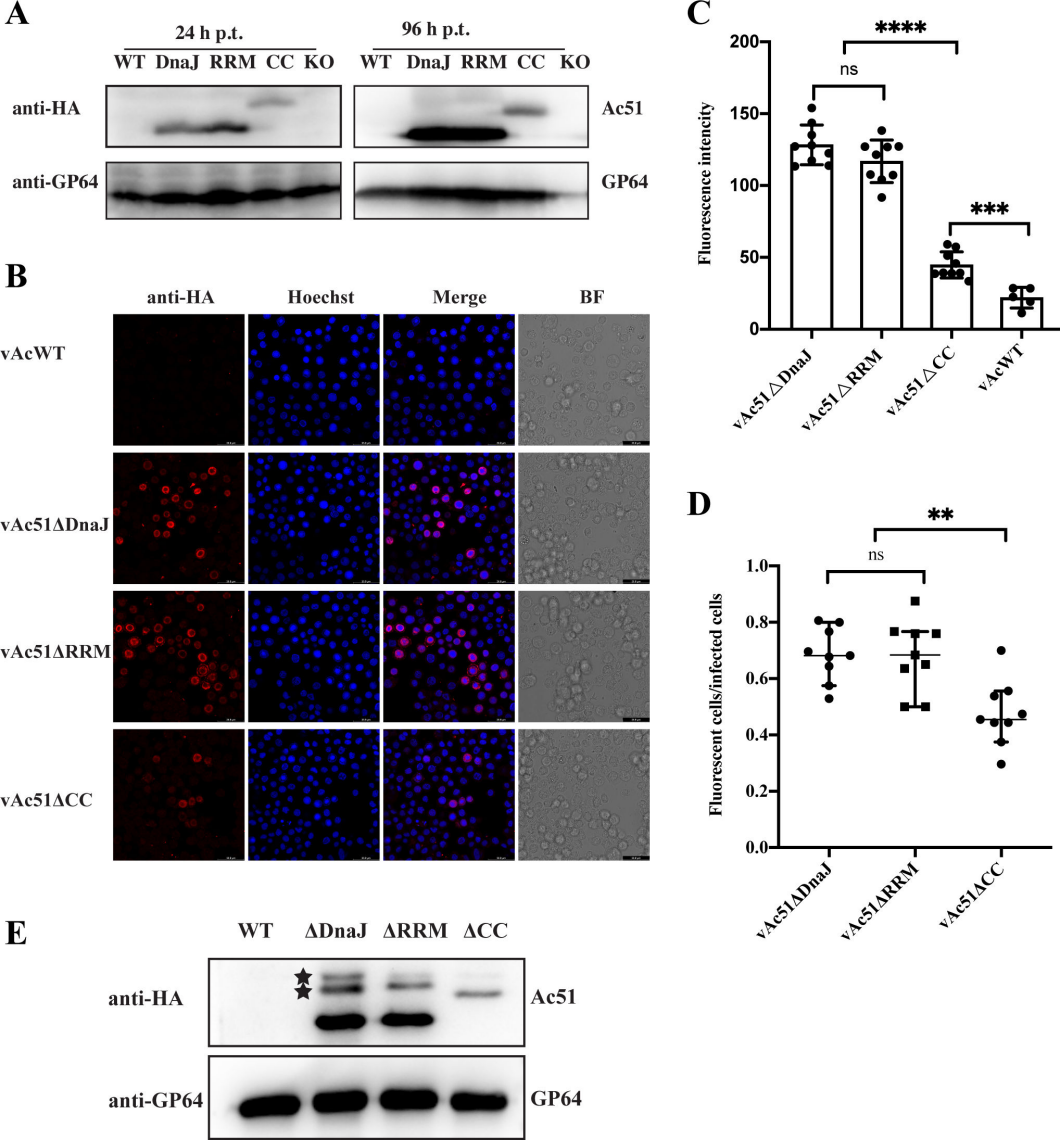
Effect of different domain deletion on the expression and localization of Ac51 in infected cells and virions. (**A**) Expression of truncated Ac51 in infected cells by western blot analysis. Sf9 cells were transfected with 1 µg of bacmid DNA of vAcWT, vAc51ΔDnaJ, vAc51ΔRRM, vAc51ΔCC, or vAc51KO. Cells were collected at 24 and 96 h p.t. for western blot analysis. Truncated Ac51 was detected using an anti-HA antibody, and GP64 was detected as the loading control. (**B**) Expression of truncated Ac51 in infected cells at 72 h p.t. by an immunofluorescence assay. Sf9 cells transfected with 1 µg of bacmid DNA of vAcWT, vAc51ΔDnaJ, vAc51ΔRRM, or vAc51ΔCC were fixed at 72 h p.t. The cells were probed with the anti-HA antibody, followed by a secondary antibody conjugated with Alexa Fluor Plus 555. Hoechst 33342 was used for nuclear staining. Bright field (BF) images were also captured. (**C**) The fluorescence intensity of truncated Ac51 in (**B**) was quantitatively analyzed. ImageJ software was used to analyze the fluorescence intensity of truncated Ac51 in nine randomly selected fields. The same analysis was performed on five randomly selected fields in vAcWT-infected cells. (**D**) The ratio of fluorescent cells to infected cells in (**B**) was determined in the nine randomly selected fields. (**E**) Localization of truncated Ac51 in BVs. BVs of the recombinant viruses were purified from the supernatant and analyzed by western blot analysis. Two nonspecific protein bands were indicated by asterisks.

Subsequently, we investigated the impact of the CC motif deletion on Ac51 accumulation with an anti-HA antibody, which is more sensitive and specific than the anti-Ac51 antibody. Sf9 cells were transfected with bacmid DNA of the recombinant viruses, and truncated Ac51 was detected using anti-HA western blot analysis. Our results indicated that deletion of the CC motif led to reduced abundance of Ac51 in infected cells compared to Ac51ΔDnaJ and Ac51ΔRRM at both 24 and 96 h p.t. (Fig. 2A). By an anti-HA immunofluorescence assay, we found that in cells infected with vAc51ΔCC, the fluorescence intensity was noticeably weaker, and the number of fluorescent cells was significantly lower compared to cells infected with vAc51ΔDnaJ or vAc51ΔRRM (Fig. 2B), which further confirmed the lower abundance of Ac51ΔCC in the cells. Using ImageJ software, the fluorescence intensity of truncated Ac51 in nine randomly selected fields was analyzed. Meanwhile, the same analysis was performed on five randomly selected fields infected with vAcWT as a control. The results showed no significant difference in the fluorescence intensity between Ac51ΔDnaJ and Ac51ΔRRM, while the fluorescence intensity of Ac51CC was approximately one-third of the other two (Fig. 2C). The ratio of fluorescent cells to infected cells was also analyzed. Cell nuclei were stained with Hoechst 33342, and the nucleus morphology of infected cells was different from that of uninfected cells, which were used to recognize infected cells. The results indicated that approximately 68% of infected cells exhibited fluorescence in vAc51ΔDnaJ- or vAc51ΔRRM-infected cells, whereas only 47% of cells exhibited fluorescence in vAc51CC-infected cells (Fig. 2D).

Immunofluorescence assay with an anti-Ac51 antibody showed that the truncated Ac51 was predominantly distributed in the nucleus, with a small proportion also distributed in the cytoplasm, showing no significant difference compared to the full-length Ac51 (Fig. S2). This result suggested that the deletion of any domain did not affect the subcellular localization of Ac51. Since Ac51 is a BV structural protein, the localization of truncated Ac51 on viral particles was further determined. BVs were purified from the supernatant of infected cells and analyzed by western blot analysis. The results indicated that all three truncated Ac51 variants, Ac51ΔDnaJ, Ac51ΔRRM, and Ac51CC, were detectable on BVs, but the abundance of Ac51ΔCC was significantly lower than that of Ac51ΔDnaJ and Ac51ΔRRM (Fig. 2E). GP64 was detected as a loading control in this analysis. Collectively, these results demonstrated that deletion of the CC motif led to a significant reduction in the protein abundance both in infected cells and on virions, which may be responsible for the significantly impaired BV production when the CC motif was deleted.

### Screening proteins that interact with Ac51 by immunoprecipitation-mass spectrometry

To elucidate the functional mechanism of Ac51 and reveal the protein-protein interaction network involved in nucleocapsid transport, immunoprecipitation-mass spectrometry (IP-MS) was employed to identify proteins that interact with Ac51. Sf9 cells were infected with vAcWT, collected at 48 hours post-infection (h p.i.), and subjected to the IP-MS with an anti-Ac51 antibody. By mapping the mass spectrometry data onto the viral and host cell proteomes, the potential interacting partners of Ac51 were revealed. In this study, we focused on the viral interacting partners. Table 1 shows the most prominent viral proteins immunoprecipitated with Ac51, including Ac132, Ac66, and ME53. Previous studies have highlighted the significance of these proteins in viral replication and transport processes. Ac66 was previously demonstrated to be one of the three proteins promoting the efficient nuclear egress of nucleocapsids. ME53 may be involved in the budding process of viral nucleocapsids from the cytoplasmic membrane to form mature BVs (34, 35). Ac132 is essential for BV production and may play a role in the retrograde transport of the nucleocapsids into the nucleus to initiate viral replication (36, 37).

**TABLE 1:** Most prominent AcMNPV proteins co-immunoprecipitated with Ac51 and identified by mass spectrometry^*a*^

Accession no.	Sequence coverage (%) (Ac51IP)	Number of peptides (Ac51IP)	Ratio (Ac51IP/IgGIP)	Mass (kDa)	Description
AIU56950.1	60	21	394.34	37.5	**AcOrf-51**
AIU57071.1	52	11	36.15	25.1	**AcOrf-132**
ANN45851.1	51	42	24.74	94.0	**Orf-66 peptide**
QZX57634.1	11	2	9.13	95.3	Orf-120
ANN45808.1	14	8	8.91	80.0	Copia-like envelope protein
ANN45864.1	18	2	8.82	12.2	Orf-79 peptide
AIU57053.1	20	2	7.42	18.4	AcOrf-93
QZX57567.1	32	8	4.49	32.1	pcna
ANN45660.1	38	19	4.25	60.7	Viral capsid-associated protein
QZX57558.1	14	6	4.19	47.5	p47
QZX57653.1	39	15	3.72	52.7	**ME53**

^*a*^ Bold formatting indicates proteins that are common to both Tables 1 and 2.

**TABLE 2:** Most prominent AcMNPV proteins Co-IP with Ac66 and identified by MS^*a*^

Accession no.	Sequence coverage (%) (FlagIP)	Number of peptides (FlagIP)	Ratio (FlagIP/IgGIP)	Mass (kDa)	Description
ANN45924.1	28	22	#N/A^*b*^	52,660	**ME53**
AIU56950.1	42	32	#N/A	37,566	**AcOrf-51**
AAA66756.1	12	6	#N/A	61,368	Chitinase
QZX57613.1	6	9	#N/A	143,099	Helicase
AAA66704.1	21	6	#N/A	30,567	AcOrf-74 peptide
QZX57607.1	18	6	#N/A	38,933	vp39
ANN45851.1	**43**	67	892.14	93,973	**Orf-66 peptide**
AIU57071.1	39	20	230.58	25,136	**AcOrf-132**
ANN45927.1	19	11	20.80	55,431	Early 49 Daa protein
AAA46685.1	79	7	13.61	8,653	V-ubi (ORF 3)

^*a*^ Bold formatting indicates proteins that are common to both Tables 1 and 2.

^*b*^ #N/A indicates missing values, where the corresponding protein was exclusively identified in the experimental group (Flag-IP).

### Ac51 interacted and colocalized with Ac66 in AcMNPV-infected cells

**Fig 3:**
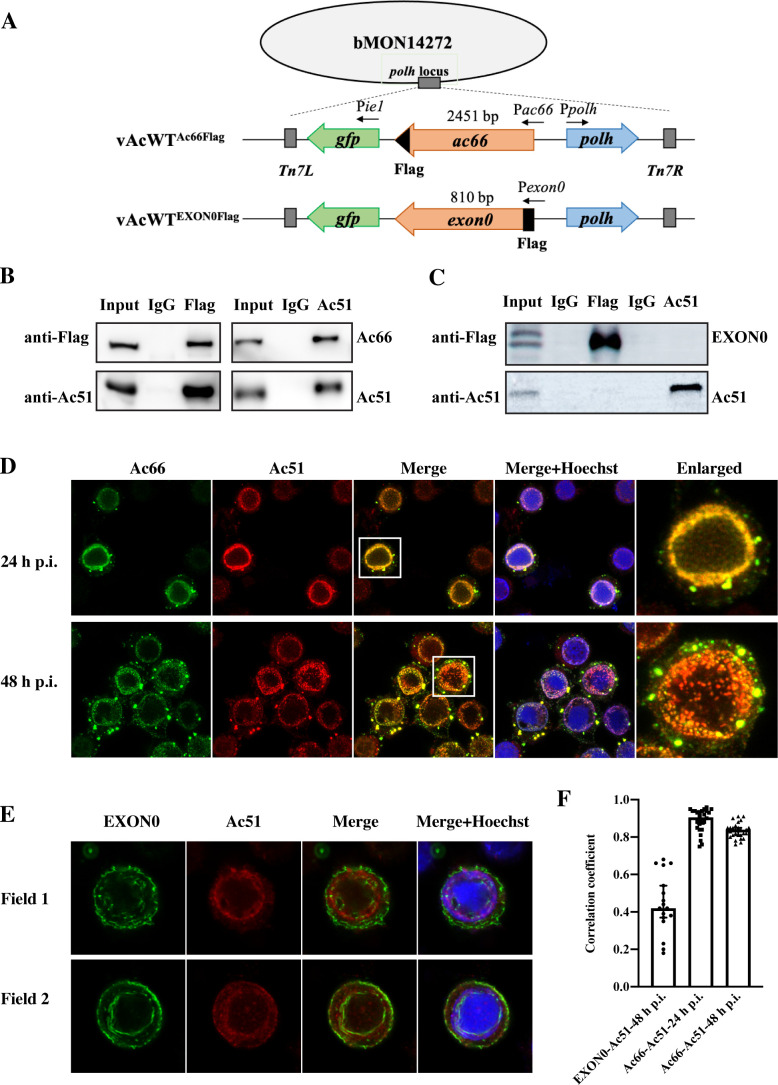
The interaction analysis between Ac51 and Ac66 and between Ac51 and EXON0. (**A**) Schematic diagram of the two recombinant viruses expressing Flag-tagged Ac66 or EXON0. (**B and C**) Interaction analysis between Ac51 and Ac66 (**B**) or between Ac51 and EXON0 (**C**) by Co-IP assay. Sf9 cells were infected with vWT^Ac66Flag^ or vWT^EXON0Flag^ at a multiplicity of infection (MOI) of 5 TCID_50_/mL and were collected at 48 h p.i. The Co-IP assay was conducted using the anti-Flag antibody or the anti-Ac51 antibody, followed by western blot analysis. (**D and E**) Colocalization analysis between Ac51 and Ac66 (**D**) or between Ac51 and EXON0 (**E**) using immunofluorescence assay. Sf9 cells infected with vWT^Ac66Flag^ or vWT^EXON0Flag^ were fixed at the indicated time point. The anti-Ac51 antibody was used to probe for Ac51, while the anti-Flag antibody was used to probe for Ac66 and EXON0. The cells within the box were magnified. (**F**) Quantitative analysis of the colocalization between Ac51 and Ac66 at 24 and 48 h p.t. and between Ac51 and EXON0 at 48 h p.i. by ImageJ software. The Pearson correlation coefficient (PCC) was utilized as a measure of colocalization strength. Infected cells were randomly selected. The number of cells analyzed (*N*) was 26 and 27 for vWT^Ac66Flag^ at 24 and 48 h p.i., respectively, while *n* was 17 for vWT^EXON0Flag^.

Since Ac51, Ac66, and EXON0 are involved in the nuclear egress of nucleocapsids, and the interaction between EXON0 and Ac66 has been proved (31), here, we examined the interaction between Ac51 and Ac66/EXON0 by co-immunoprecipitation (Co-IP) assay. Sf9 cells were infected with vWT^Ac66Flag^ or vWT^EXON0Flag^ (Fig. 3A) and subjected to the Co-IP. As depicted in Fig. 3B, the Co-IP assay revealed that Ac51 could be co-immunoprecipitated by Ac66, and reciprocally, Ac66 could also be co-immunoprecipitated by Ac51. This result demonstrated that Ac51 and Ac66 interact with each other in infected cells, indicating a potential functional association between these two proteins. On the other hand, the Co-IP assay showed that Ac51 and EXON0 could not be immunoprecipitated by each other (Fig. 3C), indicating that Ac51 does not interact with EXON0 in infected cells.

To confirm the results above, we further examined the colocalization between Ac51 and Ac66/EXON0 in infected cells. Sf9 cells were infected with vWT^Ac66Flag^, fixed at 24 and 48 h p.i., and subjected to the immunofluorescence assay. Our results showed that Ac66 and Ac51 were colocalized in both the VS and ring zone within the nucleus, Ac66 displayed a dot distribution pattern in the cytoplasm, and Ac51 colocalized with these dots (Fig. 3D). The Pearson correlation coefficient (PCC) was determined to be 0.90 and 0.84 at 24 and 48 h p.i., respectively (Fig. 3F). In addition, Sf9 cells infected with vWT^EXON0Flag^ were fixed at 48 h p.i. and subjected to the immunofluorescence assay. The results revealed a different distribution pattern between Ac51 and EXON0, and no apparent colocalization was observed (Fig. 3E). The PCC was determined to be 0.42 (Fig. 3F). These findings confirm that Ac51 interacts and colocalizes with Ac66—but not EXONO—in infected cells, providing evidence for a functional relationship between Ac51 and Ac66 in promoting the nuclear egress of nucleocapsids.

### *ac51* Deletion diminished the nuclear localization of Ac66 in infected cells

**Fig 4:**
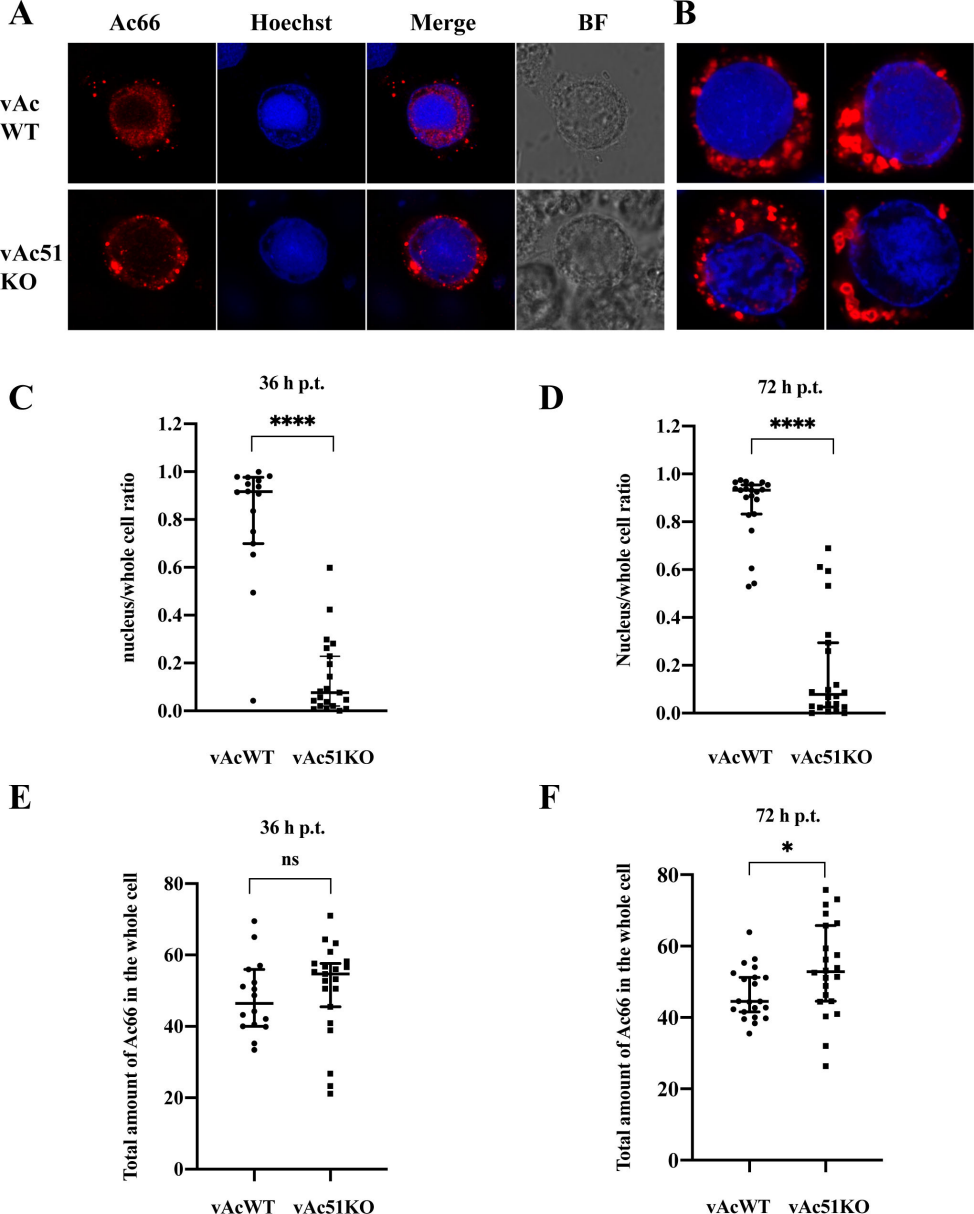
Effect of *ac51* deletion on the nuclear import of Ac66. (**A**) Subcellular distribution of Ac66 in vAc51KO- or vAcWT-infected cells at 72 h p.t. Sf9 cells were co-transfected with pUC18-Ac66Flag and bacmid DNA of vAc51KO or vAcWT and fixed at 36 h p.t. and 72 h p.t. Ac66 was detected with the anti-Flag antibody, followed by probing with the secondary antibody conjugated with Alexa Fluor Plus 647. (**B**) Different distribution pattern of Ac66 in vAc51KO-infected cells at 72 h p.t. was shown. (**C and D**) The nuclear proportion of Ac66 was analyzed at 36 h p.t. (**C**) and 72 h p.t. (**D**). (**E and F**) Fluorescence intensity of Ac66 was analyzed at 36 h p.t. (**E**) and 72 h p.t. (**F**). Infected cells were randomly selected, and nuclear proportion and the total amount of Ac66 in the whole cell were analyzed by measuring the fluorescence intensity of Ac66 using ImageJ software. The number of cells analyzed (***N***) was 16 and 21 for vAc51KO and vAcWT at 36 h p.t., respectively, and *n* was 21 and 22 for vAc51KO and vAcWT at 72 h p.t.

In order to investigate how Ac51 cooperated with Ac66 to facilitate the nuclear export of nucleocapsids, the subcellular localization of Ac66 was examined in cells infected with vAc51KO. Sf9 cells were co-transfected with pUC18-Ac66Flag and bacmid DNA of vAc51KO or vAcWT, fixed at 36 h p.t. and 72 h p.t. and subjected to the immunofluorescence assay. Ac66 was predominantly localized within the nucleus in cells infected with vAcWT and showed a dot distribution pattern in the cytoplasm at 36 h p.t. and 72 h p.t. However, in vAc51KO-infected cells, Ac66 exhibited a predominant cytoplasmic localization at 36 h p.t. and 72 h p.t. Images from 72 h p.t. were shown in Fig. 4A. Moreover, Ac66 was abnormally aggregated into punctate structures in the cytoplasm of vAc51KO-infected cells (Fig. 4B). Subsequently, randomly selected infected cells were analyzed using ImageJ software to determine the nuclear proportion of Ac66 at both 36 and 72 h p.t. The results indicated that the nuclear proportion of Ac66 in vAcWT-infected cells at 36 h p.t. was approximately 0.92, whereas in vAc51KO-infected cells, it was only 0.07 when compared to the overall cellular level (Fig. 4C). Similarly, the nuclear proportion of Ac66 was 0.93 in vAcWT-infected cells at 72 h p.t., while it decreased to 0.08 in vAc51KO-infected cells when compared to the whole cell level (Fig. 4D). Further analysis of Ac66 fluorescence intensity by ImageJ software indicated comparable abundance of Ac66 in vAcWT- or vAc51KO-infected cells at 36 h p.t. (Fig. 4E). The abundance of Ac66 was slightly higher in vAc51KO-infected cells compared to that in vAcWT-infected cells (*P* = 0.04; Fig. 4F), which may be attributed to the assembly of some Ac66 into nucleocapsids to form BVs. Taken together, these results demonstrated that Ac51 is necessary for the proper nuclear localization of Ac66 during virus infection, suggesting that Ac51 likely mediates the nuclear import of Ac66 to facilitate the nuclear egress of nucleocapsids and virus replication.

### Effect of the deletion of specific domains in Ac51 on the interaction between Ac51 and Ac66

**Fig 5:**
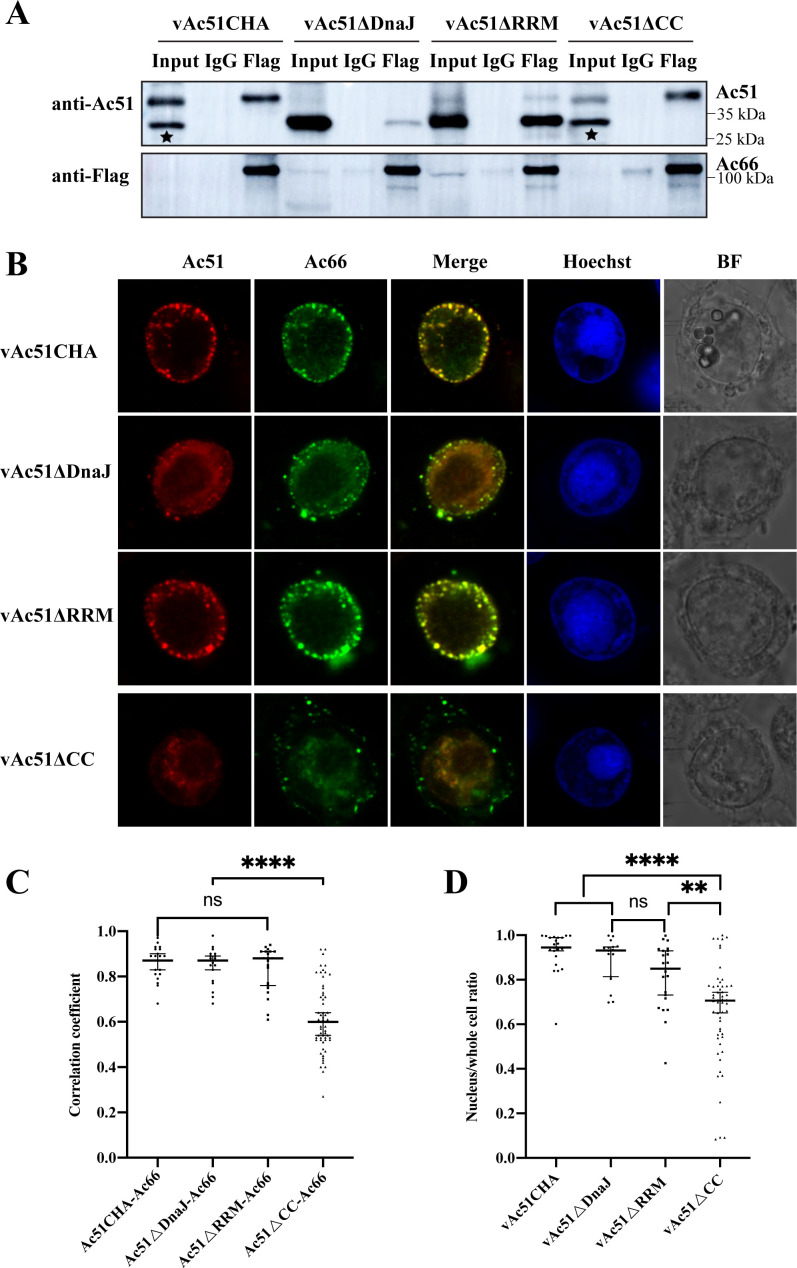
Interaction analysis between truncated Ac51 with Ac66 determined by Co-IP and immunofluorescence assays. (**A**) Interaction analysis between truncated Ac51 with Ac66 was performed using Co-IP assay. Sf9 cells were co-transfected with bacmid DNA of vAc51ΔDnaJ, vAc51ΔRRM, or vAc51ΔCC, along with pUC18-Ac66Flag, and collected at 90 h p.t. Additionally, Sf9 cells co-transfected with bacmid DNA of vAc51CHA and pUC18-Ac66Flag were collected at 48 h p.t. The cell lysate was subjected to a Co-IP assay with anti-Flag or IgG magnetic beads. Asterisks were used to indicate non-specific protein bands. (**B**) Colocalization of truncated Ac51 with Ac66 in infected cells. Sf9 cells were treated similarly to the Co-IP assay and fixed at 72 h p.t. Ac51CHA was probed with an anti-Ac51 antibody, while truncated Ac51 was probed with an anti-HA antibody. Ac66 was probed with an anti-Flag antibody. Cells were then incubated with the goat anti-mouse secondary antibody conjugated with Alexa Fluor Plus 647 or the goat anti-rabbit secondary antibody conjugated with Alexa Fluor Plus 555. (**C**) PCC between truncated Ac51 and Ac66. Randomly selected infected cells (vAc51CHA, *n* = 23; vAc51ΔDnaJ, *n* = 20; vAc51ΔRRM, *n* = 21; vAc51ΔCC, *n* = 59) were analyzed using ImageJ software, which calculates the PCC between proteins. (**D**) The nuclear proportion of Ac66 in vAc51ΔDnaJ-, vAc51ΔRRM-, or vAc51ΔCC-infected cells. Randomly selected infected cells (vAc51CHA, *n* = 23; vAc51ΔDnaJ, *n* = 17; vAc51ΔRRM, *n* = 23; vAc51ΔCC, *n* = 54) were analyzed using ImageJ software to determine the ratio of nuclear proportion vs whole cells.

We next determined the conserved domains within Ac51 that mediate its interaction with Flag-tagged-Ac66. Sf9 cells were co-transfected with bacmid DNA of vAc51ΔDnaJ, vAc51ΔRRM, or vAc51ΔCC along with pUC18-Ac66Flag and collected at 90 h p.t. Additionally, Sf9 cells co-transfected with bacmid DNA of vAc51CHA and pUC18-Ac66Flag were collected at 48 h p.t. The cell lysate was subjected to Co-IP assays with anti-Flag or IgG magnetic beads. The results demonstrated that all three mutants, Ac51ΔDnaJ, Ac51ΔRRM, and Ac51ΔCC, were co-immunoprecipitated by Ac66 to some extent (Fig. 5A). Ac51ΔRRM showed a significant level of co-immunoprecipitation with Ac66, indicating that the deletion of the RRM did not significantly affect the interaction. In contrast, only a low level of Ac51ΔDnaJ was co-immunoprecipitated with Ac66 compared to its high expression level in whole cell lysate (input), suggesting that deletion of the DnaJ domain greatly impaired the interaction between Ac51 and Ac66 *in vitro*. Although present at a low expression level, some Ac51ΔCC could be co-immunoprecipitated by Ac66. There were non-specific protein bands in the input samples using the anti-Ac51 antibody, which were indicated with asterisks.

Furthermore, the colocalization of truncated Ac51 and Flag-tagged Ac66 was determined by immunofluorescence assay. The results revealed that Ac51CHA (probed with an anti-Ac51 antibody), Ac51ΔDnaJ, and Ac51ΔRRM (probed with an anti-HA antibody) exhibited considerable colocalization with Ac66 in infected cells. However, Ac51ΔCC (probed with an anti-HA antibody) showed a relatively lower degree of colocalization with Ac66 (Fig. 5B). Statistical analysis of the PCC further supported these observations, with Ac51CHA, Ac51ΔDnaJ, Ac51ΔRRM, and Ac51ΔCC exhibiting correlation coefficients of 0.87, 0.87, 0.88, and 0.60, respectively (Fig. 5C). Moreover, the nuclear proportion of Ac66 in cells infected with vAc51CHA, vAc51ΔDnaJ, vAc51ΔRRM, or vAc51ΔCC was assessed using ImageJ software, and the results were 0.94, 0.93, 0.85, and 0.70, respectively (Fig. 5D). In summary, these results indicated that although the deletion of any domain did not completely abolish the interaction between Ac51 and Ac66, the DnaJ domain appears to be crucial for maintaining their strong binding affinity *in vitro*, and deletion of the CC motif significantly reduced its colocalization with Ac66 and impacted the nuclear localization efficiency of Ac66.

### ME53 and Ac132 interacted and colocalized with both Ac51 and Ac66 in infected cells

**Fig 6:**
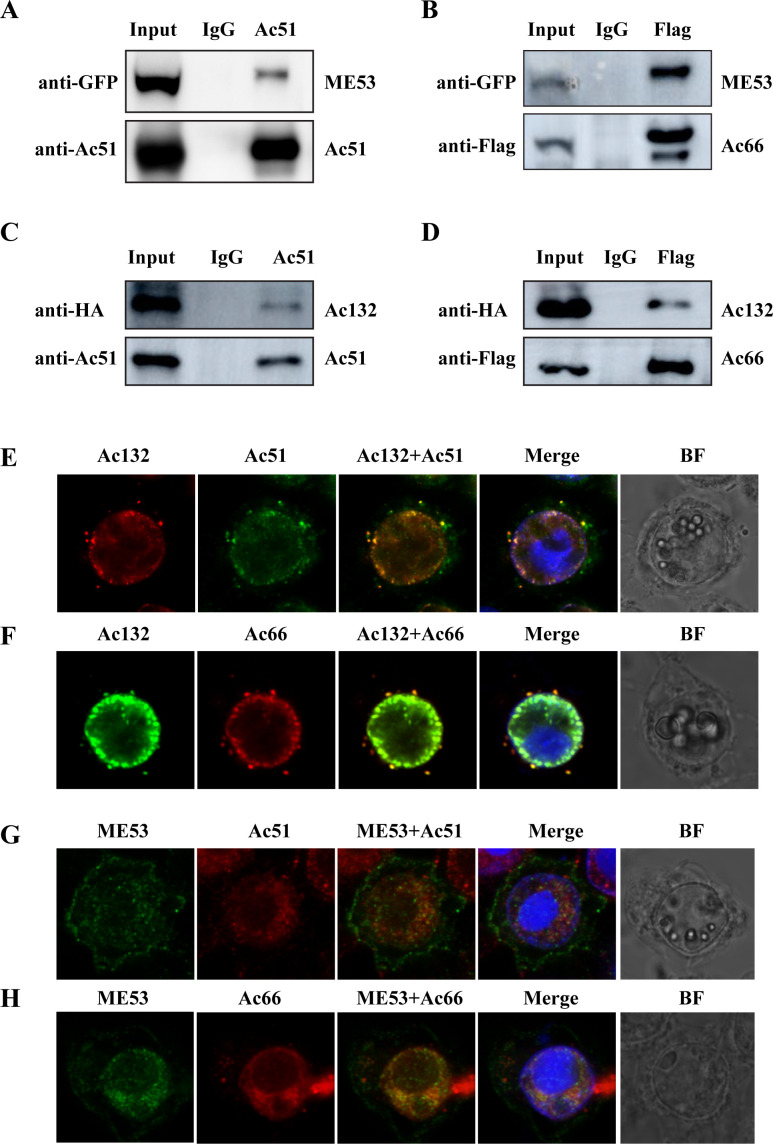
Interaction and colocalization analysis between ME53 and Ac66, ME53 and Ac51, Ac132 and Ac51, and Ac132 and Ac66. (**A and B**) Interaction between ME53 and Ac51/Ac66 by Co-IP assay. Sf9 cells were transfected with pIB-ME53GFP. At 24 h p.t., cells were infected with vAcWT (**A**) or vAcWT^Ac66flag^ (**B**) at a multiplicity of infection (MOI) of 5 TCID_50_/mL. At 48 h p.i., the cells were collected and subjected to the Co-IP assay using the anti-Ac51 antibody (**A**) or anti-Flag antibody (**B**). IgG from correspondent species was used as the negative control. All samples from the Co-IP assay were analyzed by western blot analysis with the respective antibodies indicated in the figures. (**C and D**) Interaction analysis between Ac132 and Ac51/Ac66 by Co-IP assay. Sf9 cells infected with vAc132HA (**C**) or co-infected with vAc132HA and vAcWT^Ac66flag^ (**D**) at an MOI of 5 TCID_50_/mL were collected at 48 h p.i. and subjected to Co-IP assay with the anti-Ac51 antibody (**C**) or anti-Flag antibody (**D**). IgG from correspondent species was used as the negative control. All samples from the Co-IP assay were analyzed by western blot analysis using the respective antibodies indicated in the figures. (**E and F**) Colocalization analysis between Ac132 and Ac51 (**F**) or between Ac132 and Ac66 (**F**). Sf9 cells treated the same as in the Co-IP assay were subjected to the immunofluorescence assay at 48 h p.i. Ac132, Ac51, and Ac66 were probed with the anti-HA, anti-Ac51, and anti-Flag antibodies, respectively. (**G and H**) Colocalization analysis between ME53 and Ac51 (**G**) or between ME53 and Ac66 (**H**). Sf9 cells co-transfected with pUC18-ME53Myc and bacmid DNA of vAcWT (**G**) or vAcWT^Ac66Flag^ (**H**) were subjected to the immunofluorescent assay at 48 h p.t. ME53, Ac51, and Ac66 were probed with the anti-Myc, anti-Ac51, and anti-Flag antibodies, respectively.

We also performed screening by IP-MS to identify proteins that interact with Ac66. The most prominent viral proteins immunoprecipitated with Ac66 are shown in Table 2. An interesting finding is that both ME53 and Ac132 were also present as interacting proteins with Ac66. Moreover, previous studies have demonstrated that both ME53 and Ac132 are nucleocapsid proteins, but neither deletion affected nucleocapsid assembly (35–37), similar to the phenomena observed in *ac66*/*ac51* knockout virus. To validate the interaction between ME53 and Ac66, ME53 and Ac51, Ac132 and Ac51, and Ac132 and Ac66, a Co-IP assay was performed. Sf9 cells were transfected with pIB-ME53GFP and subsequently infected with vAcWT^Ac66Flag^ or vAcWT. The cells were collected at 48 h p.i. for analysis. The Co-IP assay confirmed that ME53 could be immunoprecipitated by both Ac51 (Fig. 6A) and Ac66 (Fig. 6B), demonstrating the interaction between ME53 and Ac51/Ac66. Similarly, Sf9 cells infected with vAc132HA alone or co-infected with vAc132HA and vAcWT^Ac66Flag^ were collected at 48 h p.i. The results indicated that Ac132 could also be immunoprecipitated by both Ac51 (Fig. 6C) and Ac66 (Fig. 6D), confirming the interaction between Ac132 and Ac51/Ac66. To examine the colocalization of these proteins, an immunofluorescent assay was conducted. Sf9 cells were treated the same as described above. The results revealed that Ac132 colocalized with Ac51 and Ac66 in both the nucleus and cytoplasm. In the cytoplasm, Ac132 formed foci with Ac51 and Ac66 (Fig. 6E and F). Additionally, Sf9 cells co-transfected with pUC18-ME53Myc and bacmid DNA of vAcWT^Ac66Flag^ or vAcWT were subjected to the immunofluorescent assay. The results showed that ME53 colocalized with both Ac51 and Ac66 in the ring zone (Fig. 6G and H). Collectively, these findings provide further evidence of the interactions between ME53 and Ac66, ME53 and Ac51, Ac132 and Ac51, and Ac132 and Ac66.

### Interaction analysis among Ac51, Ac66, ME53, and Ac132 by molecular docking

**Fig 7:**
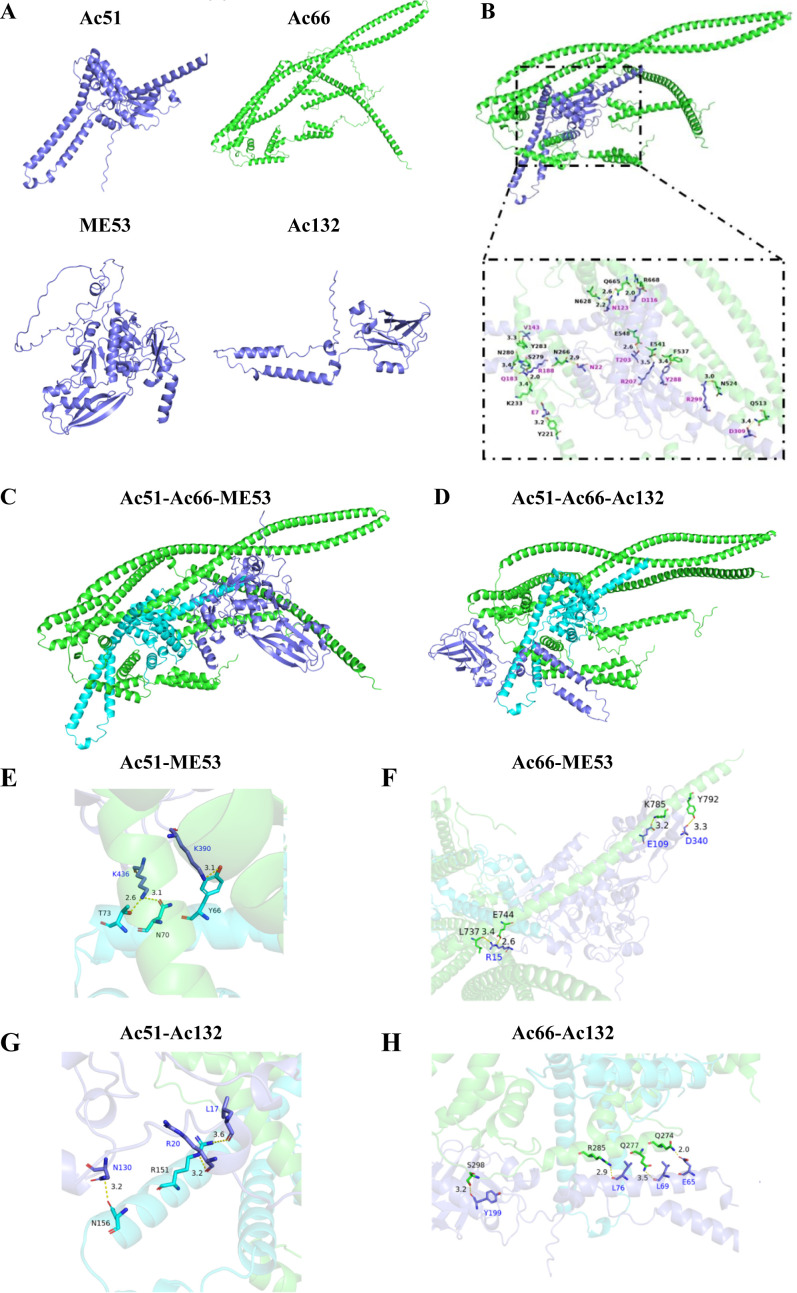
Interaction analysis between Ac51, Ac66, ME53, and Ac132 by molecular docking analysis. (**A**) The 3D structures of Ac51, Ac66, ME53, and Ac132 were predicted by AlphaFold 2 software. (**B**) The 3D structure of the Ac51-Ac66 complex. Molecular docking of Ac51 and Ac66 was carried out using HDOCK software, resulting in the prediction of a complex structure. The hydrogen bonds and the involved amino acid residues between Ac51 and Ac66 were analyzed using PyMOL software and showed. (**C and D**) The 3D structure of the complex formed between ME53/Ac132 and the Ac51-Ac66 complex. The 3D structure of the protein complex was generated using HDOCK software. (**E–H**) The hydrogen bonds and the involved amino acid residues between ME53 and Ac51 (**E**), ME53 and Ac66 (**F**), Ac132 and Ac51 (**G**), and Ac132 and Ac66 (**H**) were analyzed using PyMOL software and depicted in the figures.

Molecular docking is a computational technique used to predict the binding affinities and orientations of ligands when interacting with proteins. Here, we applied molecular docking to further elucidate the interaction mechanism between Ac51 and Ac66. The predicted three-dimensional (3D) structures of proteins were obtained using the AlphaFold 2 software (Fig. 7A). Molecular docking and data analysis were performed using the HDOCK and PyMOL software. The results revealed an optimal docking score of −336.67 for the Ac51-Ac66 interaction, with a high confidence score of 0.9766, indicating a reliable docked complex model. This result suggested that Ac51 and Ac66 can bind together effectively, with Ac51 inserting into the cavity of the Ac66 protein, resembling a dovetail joint structure (Fig. 7B). In the docked complex, a total of 14 hydrogen bonds were predicted to be formed between the two proteins. Key amino acid residues predicted to be involved in hydrogen bonding, along with the bond lengths, are listed in Table 3. Notably, amino acids in the Ac51 protein that are supposed to form strong hydrogen bonds with Ac66 include D116, N123, N123, and Q183, with hydrogen bond distances of 2.0, 2.2, 2.6, and 2.0 Å, respectively.

**TABLE 3:** Amino acid residues involved in hydrogen bonding between proteins along with the hydrogen bond distances^*a*^

Amino acid residues in Ac51	Amino acid residues in Ac66	Amino acid residues in ME53	Amino acid residues in Ac132	Hydrogen bond distance (Å)
E7	Y221	–	–	3.2
R188	K223	–	–	3.4
N22	N266	–	–	2.9
Q183	S279	–	–	2
Q183	N280	–	–	3.4
V143	Y283	–	–	3.3
D309	Q513	–	–	3.4
R299	N524	–	–	3
Y288	F537	–	–	3.4
R207	E541	–	–	3.5
T203	E548	–	–	2.6
N123	N628	–	–	2.2
N123	Q665	–	–	2.6
D116	R668	–	–	2.0
E7	Y221	–	–	3.2
Y66	–	K390	–	3.1
N70	–	K436	–	3.1
T73	–	K436	–	2.6
–	L737	R15	–	3.4
–	E744	R15	–	2.6
–	K785	E109	–	3.2
–	Y792	D340	–	3.3
R151	–	–	L17	3.6
R151	–	–	R20	3.2
N156	–	–	N130	3.2
–	Q274	–	E65	2.0
–	Q277	–	L69	3.5
–	R285	–	L76	2.9
–	S298	–	Y199	3.2

^*a*^ –, not applicable.

Subsequently, we investigated the interaction modes of ME53/Ac132 with the Ac51-Ac66 complex. ME53 and Ac132 were individually docked onto the Ac51-Ac66 complex. The docking scores for ME53 and Ac132 with the Ac51-Ac66 complex were calculated as −322.47 and −259.67, respectively. These scores, along with the confidence scores of 0.9692 for ME53 and 0.8997 for Ac132, indicated a high likelihood of the docked complex models. The predicted 3D structures of the complex formed between ME53/Ac132 and Ac51-Ac66 complex are shown in Fig. 7C and D. The docking results showed that ME53 and Ac132 were predicted to be bound to different regions of the Ac51-Ac66 complex, suggesting distinct binding modes for these proteins. Predicted hydrogen bonds formed between ME53 and Ac51, ME53 and Ac66, Ac132 and Ac51, and Ac132 and Ac66 were illustrated in panels E, F, G, and H, respectively. The amino acid residues involved in these hydrogen bonding, along with the bond lengths, are listed in Table 3. These results confirmed that ME53 and Ac132 interact effectively with the Ac51-Ac66 complex, consistent with our experimental results (Fig. 6).

### *ac51* Knockout did not affect the subcellular localization of ME53 and Ac132 in infected cells

**Fig 8:**
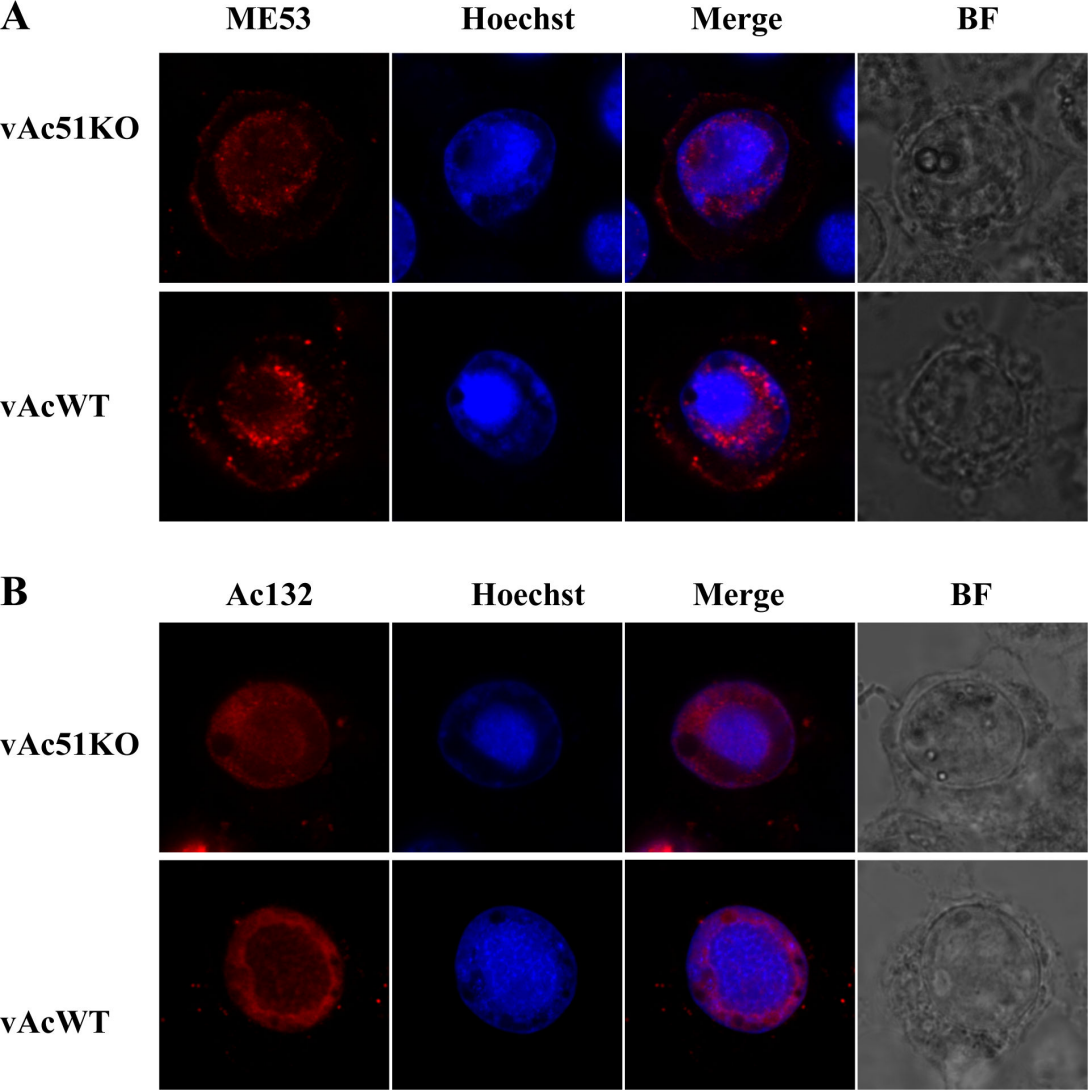
Subcellular localization of ME53 (**A**) and Ac132 (**B**) in vAc51KO-infected cells. Sf9 cells were co-transfected with bacmid DNA of vAc51KO or vAcWT along with pUC18-ME53Myc or pUC18-Ac132HA and subjected to the immunofluorescence assay at 72 h p.t. ME53 was detected with an anti-Myc antibody, and Ac132 was probed with an anti-HA antibody.

Since Ac51 is required for the nuclear localization of Ac66, we further examined the effect of *ac51* knockout on the subcellular localization of ME53 and Ac132. Sf9 cells were co-transfected with bacmid DNA of vAc51KO or vAcWT together with pUC18-ME53Myc or pUC18-Ac132HA and subjected to the immunofluorescence assay. As shown in Fig. 8A, in vAc51KO-infected cells, ME53 was predominantly localized in the nucleus and cytoplasmic membrane of cells, similar to its localization in vAcWT-infected cells. As shown in Fig. 8B, Ac132 was predominantly distributed in the nucleus of infected cells, and this localization was not affected by *ac51* knockout. These results indicated that the knockout of *ac51* did not appear to affect the subcellular localization of ME53 and Ac132 and specifically exerted its function on the nuclear import of Ac66.

## DISCUSSION

The nuclear egress of AcMNPV nucleocapsids is a critical step in progeny virus production, yet its underlying mechanism and the protein-protein interactions involved are not fully understood. In our previous study, we identified Ac51 as one of the proteins involved in this process, but its specific mechanism is unclear. In the present study, we made significant discoveries regarding the role of Ac51 in efficient virus production. Our study elucidated the functional importance of the three conserved domains of Ac51 in virus replication. We demonstrate that Ac51 interacts with Ac66 and exhibits a high degree of colocalization with it in infected cells. Their interaction mechanism was revealed. Subsequent investigation revealed that Ac51 played a crucial role in mediating the nuclear localization of Ac66. Our study also uncovered additional interaction and colocalization between two other nucleocapsid proteins, ME53 and Ac132, with both Ac51 and Ac66.

### The CC motif in Ac51 plays a critical role in the accumulation of Ac51 within infected cells and efficient virus multiplication

Our results showed that Ac51 variants with deletion in the DnaJ domain or RRM still exhibited abundant expression in infected cells (Fig. 1E and Fig. 2). However, when the CC motif was deleted, a significant decrease in the abundance of Ac51 within infected cells was observed (Fig. 1E and Fig. 2). These results indicate that the CC motif is crucial for the accumulation of Ac51 in infected cells. Plaque assay and virus growth curve analysis showed that vAc51ΔCC exhibited the weakest replication ability compared to vAc51ΔDnaJ and vAc51ΔRRM (Fig. 1C and D). These results suggest that the low expression level of Ac51ΔCC contributed to the compromised viral replication. It is possible that the deletion of the CC motif may have altered the 3D structure of the Ac51 protein, potentially affecting its stability. Alternatively, since the CC motifs generally mediate protein-protein interactions (38, 39), deletion of the CC motif may disrupt the interaction between Ac51 and certain proteins that stabilize Ac51 within infected cells. Consequently, the absence of these stabilizing interactions could result in the degradation of Ac51. Further investigation is required to determine whether Ac66, ME53, or Ac132 is involved in maintaining the stability of Ac51.

### Possible mechanisms of the Ac51-Ac66 interaction to facilitate the nuclear egress of nucleocapsids to form BVs

Theoretically, Ac66 undergoes translocation to the nucleus where it participates in the nucleocapsid assembly, which may be important for nucleocapsid transport as a nucleocapsid protein. Indeed, it has been reported that Ac66 can be ubiquitinated by viral ubiquitin, possibly involving the assistance of the potential viral E3 ubiquitin ligase, EXON0, and this ubiquitination may serve as a signal for nucleocapsid transportation out of the nucleus (31). On the other hand, Ac66 is a structural protein that may facilitate the assembly of nucleocapsids by mediating the incorporation of other proteins, such as Ac51, ME53, Ac132, or EXON0, into nucleocapsids. The interactions between Ac66 and these proteins in the ring zone support their involvement in nucleocapsid assembly and subsequent transport. Additionally, Ac66 may have a role within the nucleus itself, promoting the nuclear egress of nucleocapsids. An interesting phenomenon was that Ac66 is prominently localized at the nuclear periphery. This suggests the potential involvement of Ac66 in assisting the passage of nucleocapsids across the nuclear membrane. A previous study has observed microfilament-mediated nuclear envelope depolymerization at the budding sites of nucleocapsids, suggesting the involvement of microfilaments in the nuclear egress of nucleocapsids (40). Interestingly, host actin and myosin peptides were identified by IP-MS in proteins immunoprecipitated with Ac66. This suggests a potential association between Ac66 and microfilaments during nucleocapsid egress, which opens up new avenues for further investigation into the intricate mechanisms underlying nucleocapsid transportation out of the nucleus.

### Amino acid regions in Ac51 that mediate its interaction with Ac66

Our study revealed that the interaction between Ac51 and Ac66 is not solely dependent on any specific conserved domain of Ac51, as the deletion of individual domains did not abolish their interaction. This suggested the presence of other crucial regions in Ac51 that mediate the interaction between Ac51 and Ac66. Indeed, the molecular docking analysis implicated a robust interaction between Ac51 and Ac66, where Ac51 was inserted into the cavity of Ac66, resembling a dovetail joint structure. Amino acid residues in Ac51, including E7, N22, D116, N123, V143, Q183, R188, T203, R207, Y288, R299, and D309, were predicted to form hydrogen bonds with Ac66, suggesting that the region spanning from D116 to R207 in Ac51 appears to be the main region mediating the interaction with Ac66. Of particular interest, D116 and N123, located in or near the DnaJ domain, form three strong hydrogen bonds with Ac66, with hydrogen bond distances of 2.0, 2.2, and 2.6 Å (Table 3). These interactions likely contribute to the high affinity between Ac51 and Ac66. When the DnaJ domain was deleted, their interaction *in vitro* was significantly weakened, as shown by the Co-IP assay (Fig. 5A). Interestingly, Ac51ΔDnaJ still exhibited a high degree of colocalization with Ac66, and the nuclear translocation of Ac66 appeared unaffected by the deletion of the DnaJ domain, compared to the deletion of the RRM (Fig. 5B and D). This may be due to the remaining weak binding between Ac51ΔDnaJ and Ac66 within infected cells.

The Co-IP assay indicated that, despite being present at low levels, some Ac51ΔCC could be immunoprecipitated by Ac66 (Fig. 5A), suggesting that Ac51ΔCC retains the ability to interact with Ac66. Furthermore, in vAc51ΔCC-infected cells, some Ac66 aggregated in the cytoplasm, forming punctate structures similar to those observed in vAc51KO-infected cells. This finding suggests that the low level of Ac51ΔCC was insufficient to transport the abundant Ac66 to the nucleus, resulting in the retention of some Ac66 in the cytoplasm. Consequently, deletion of the CC motif significantly impaired the colocalization between Ac51 and Ac66, the nuclear localization of Ac66 in infected cells, and finally virus multiplication. Taken together, it is speculated that the nuclear localization of Ac66 is crucial for virus replication, and Ac51 may exert its function by interacting with Ac66 and mediating its nuclear translocation to promote nuclear egress of nucleocapsids and virus replication.

### Mechanism of nucleocapsid transport involving the complex formation of Ac51-Ac66-ME53-Ac132

An intriguing finding from our study is the interaction among Ac51, Ac66, ME53, and Ac132 in infected cells. These interactions were confirmed by various experiments, including IP-MS, Co-IP assay, immunofluorescence assay, and molecular docking (Tables 1 and 2, Fig. 6 and 7). Moreover, the molecular docking analysis suggested that Ac132 and ME53 bound to different regions of the Ac51-Ac66 complex (Fig. 7 and Table 3). ME53 is an early and late viral protein known to function as a transcription factor. Previous studies showed that ME53 is a BV structural protein, and its deletion did not affect viral DNA replication and nucleocapsid assembly but resulted in a 3-log reduction in BV production, similar to the phenotype observed in *exon0*/*ac66*/*ac51* knockout virus. Deletion of the late promoter of the *me53* gene severely compromised virus titer, indicating its vital role during the late phase of the infection cycle when BV and ODV production occurs (35). In the present study, we demonstrated the interaction of ME53 with Ac51 and Ac66, along with their colocalization in the ring zone where BV and ODV assembled (Fig. 6), suggesting their functional association. These three proteins may interact with each other to facilitate their assembly in BVs and work together to promote the nuclear egress and subsequent transport of nucleocapsids. Additionally, a proportion of ME53 was found to be distributed in the cytoplasmic membrane (Fig. 6G and H), consistent with results in previous studies (34, 41). Another study demonstrated that ME53 colocalized with GP64 and VP39 in the cytoplasmic membrane, and its localization in this region depended on GP64 and microfilament, as *gp64* deletion or microfilament depolymerization destroyed such distribution (34). This suggested a role for ME53 in the process of nucleocapsid budding to form mature BVs. Given the interaction of ME53 with the Ac51-Ac66 complex, it is possible that they may play a role in guiding the nucleocapsids to the budding site in the cytoplasm for virion release or function during the budding process. Notably, BV production still occurs to some extent in the absence of *v-ubiquitin*/*exon0*/*ac66*/*ac51*/*me53*, suggesting a certain degree of functional redundancy among these proteins. They may assume similar roles, albeit with significantly reduced efficiency when one of them is lacking.

Ac132 is also a nucleocapsid protein (36, 37). In our previous study, we observed that deletion of the *ac132* gene also did not affect the nucleocapsid assembly, but it resulted in the absence of infectious progeny BVs. Interestingly, BV particles were detectable in the supernatant when *ac132* was deleted, but they were unable to undergo retrograde transport to enter the nucleus for replication during the second infection cycle. These findings suggest that Ac132 plays a crucial role in facilitating the retrograde transport of nucleocapsids into the nucleus (37). It is worth noting that another study reported the absence of BVs in the supernatant when *ac132* was deleted, suggesting a role for Ac132 in BV release to the extracellular environment (36). The reason for the different phenotypes observed in these two studies requires further investigation. In this study, we discovered the interaction between Ac132 and the Ac51-Ac66 complex. They predominantly colocalized in the ring zone and formed foci in the cytoplasm, with some foci appearing at the surface of the infected cells, similar to the GP64 and ME53 foci at the cell surface. This suggests their potential cooperation in nucleocapsid assembly and subsequent nucleocapsid transport. It remains to be determined whether the Ac51-Ac66 complex is required for the retrograde transport of nucleocapsids. In addition, while the deletion of *ac51* affected the nuclear localization of Ac66, it did not affect the nuclear localization of Ac132 (Fig. 8B). Furthermore, the deletion of *ac132* did not affect the subcellular localization of Ac51 and Ac66 (Fig. S3). These findings suggest that Ac132 functions in conjunction with the Ac51-Ac66 complex in the nucleus and subsequently processes. Further research is needed to fully understand the precise mechanisms by which the Ac51-Ac66-ME53-Ac132 complex contributes to nucleocapsid transport.

In conclusion, our study demonstrated that baculovirus Ac51 exerted its function on Ac66 by interacting and mediating its efficient nuclear localization to promote the nuclear egress of nucleocapsids and virus production. Our study also elucidated the possible interaction mechanism between Ac51 and Ac66. The Ac51-Ac66-ME53-Ac132 complex was further identified and may play a crucial role in coordinating the assembly, transport, and release of nucleocapsids. It remains to be investigated how the four-protein complex functions in virus replication.

## MATERIALS AND METHODS

### Cells, viruses, and bacmids

Sf9 cells were cultured in Grace’s insect cell culture medium (Supplemented, Gibco) supplemented with 10% fetal bovine serum at 27°C. DH10Bac cells, which contained bMON14272, pBAD-gbaA (encoding recombinase), and the helper plasmid pMON7124 (encoding transposase), were used for homologous recombination and transposition to generate AcMNPV recombinants. Recombinant viruses, including bAc51KO, vAc51KO, and vAcWT, were constructed in our previous study (27). bAc51KO represents a bMON1427 with the *ac51* gene deleted. vAc51KO represents an AcMNPV with the *ac51* gene deleted and *polh* and *gfp* genes inserted into the *polh* locus. vAcWT represents a wild-type control virus with *polh* and *gfp* genes inserted into the *polh* locus. vAc132HA and vAc132KO (provided by Professor Yang Kai’s lab) were constructed in another study (37). In all the experiments, the cells were allowed to adsorb the viral inocula for 1 h p.i. or 5 h p.t. at 27°C, and then the medium was replaced with fresh medium. Ultrapure bacmid DNA of viruses was extracted from bacteria using a NucleoBond BAC 100 kit (Macherey-Nagel, USA). Ultrapure plasmids were extracted from bacteria using an EndoFree Maxi Plasmid Kit (TIANGEN, DP117).

### Preparation of a polyclonal rabbit antibody against Ac51

To generate an anti-Ac51 antibody, the open reading frame (ORF) of *ac51* was synthesized and then inserted into pET-30a to produce the prokaryotic expression plasmid pET-30a-Ac51. After confirming the sequence via DNA sequencing, pET-30a-Ac51 was transformed into *Escherichia coli* strain BL21 for expression of the Ac51 protein. The expressed Ac51 protein was purified using nickel column affinity purification, and New Zealand rabbits were immunized with the purified protein to generate a polyclonal antibody against Ac51 by the GenScript Company (Nanjing, China). The serum containing the anti-Ac51 antibody was subsequently extracted and purified using the antigen-specific affinity method.

### Construction of recombinant viruses with deletion of conserved domains in Ac51

First, a fragment containing the native *ac51* promoter and its ORF with an HA tag sequence at the C terminus was amplified by PCR from bMON14272 using the primers P515 and 51rep3 (Table 4). The PCR products were then digested with *Sac* I and *Bam*H I and ligated into pUC18-SV40, resulting in the generation of pUC18-Ac51CHA-SV40. Using the site-directed mutagenesis (42) and specific primer pairs DnaJFt/DnaJRs, DnaJFt/DnaJRs, RRMFt/RRMRs, and RRMFs/RRMRt (Table 4), recombinant plasmids pUC18-Ac51ΔDnaJ-SV40 and pUC18-Ac51ΔRRM-SV40 were generated from pUC18-Ac51CHA-SV40 by deleting the DnaJ and RRM domains, respectively. The DNA sequence encoding Ac51ΔCC was amplified from bMON14272 with the primer pair P515/51dC3 (Table 4), digested with *Sac* I and *Bam*H I, and ligated to pUC18-SV40 to generate the recombinant plasmid pUC18-Ac51ΔCC-SV40. All truncated Ac51 was expressed under the Ac51 native promoter. Subsequently, the Ac51CHA-SV40, Ac51ΔDnaJ-SV40, Ac51ΔRRM-SV40, and Ac51ΔCC-SV40 fragments containing the promoter sequences were digested with *Sac* I and *Xba* I from pUC18 and ligated into pFB1-PG (43), resulting in recombinant plasmids pFB1-PG-Ac51CHA, pFB1-PG-Ac51ΔDnaJ, pFB1-PG-Ac51ΔRRM, and pFB1-PG-Ac51ΔCC. The three plasmids were transformed individually into chemically competent DH10B cells containing bAc51KO to generate recombinant viruses via Tn7-mediated transposition, and the viruses were designated vAc51CHA, vAc51ΔDnaJ, vAc51ΔRRM, and vAc51ΔCC. All of the recombinants were verified by PCR and sequencing.

**TABLE 4:** Primers used in this study^*a*^

Primer	Sequence (5′−3′)
P515	CGAGAGCTCCGCGATTGATGTGCTTCTCTTG
51rep3	CGCGGATCCTTAGGCGTAATCTGGGACGTCGTATGGGTATGTGTAAGATTTTAAAGCGTTTCTTAAATC
DnaJFt	AAAAAAAATACAGACTTGGAATCGTACGATCCATTGATTACGGT
DnaJRs	TTCCAAGTTGCAAAACCCCAAAA
DnaJFs	ATCGTACGATCCATTGATTACGGT
DnaJRt	TCCAAGTCTGTATTTTTTTTTTCCAAGTTGCAAAACCCCAAAA
RRMFt	TTGTAAAACGTCCTACAACTTTTTCCGTGGTTCAGTACTACAA
RRMRs	TTATAGTTTTAGTTTTTTGTTTA
RRMFs	TTTTCCGTGGTTCAGTACTACAA
RRMFt	AGTTGTAGGACGTTTTACAATTATAGTTTTAGTTTTTTGTTTA
51dC3	CGCGGATCCTTATGTTTTGGCCACGTTGTAG
ac66F	CGAGAGCTCTGCTTTGTTTCTTTCGTATTTAATCACG
ac66R	CGCGGATCCTTACTTATCGTCGTCATCCTTGTAATCTTCGACGTTTGGTTGAACGC
ac132F	CGAGCTCACCCGACTTGACGTCCATG
ac132R	CGCGGATCCCTAGGCGTAGTCGGGCACGTCGTAGGGGTAAAGACCTAATGGTAAAATGACGGGA
me53F	CGAGCTCACGTGACTGCTGGTTCTTATCA
me53R	CGCGGATCCTTACAGATCCTCTTCAGAGATGAGTTTCTGCTCGACATTGTTATTTACAATATTAATTAACTT

^*a*^ Nucleotide sequences with protecting bases recognized by restriction endonucleases are underlined, and nucleotide sequences encoding tags are marked with wavy lines.

### Construction of recombinant viruses expressing Flag-tagged Ac66 or Flag-tagged EXON0

Flag-tagged Ac66 or Flag-tagged EXON0 were constructed based on strategies from previous studies (31). DNA sequences containing the ORF of *ac66* and its corresponding promoter were PCR amplified from bMON1427 with Flag-encoding sequence tagged at the C-terminus of Ac66 ORF using primer pairs ac66F/ac66R (Table 4). The PCR products were digested with *Sac* I and *Bam*H I and ligated to pUC18-SV40 to generate pUC18-Ac66Flag. Then the Ac66-Flag-SV40 fragment was digested from pUC18 with *Sac* I and *Xba* I and ligated to pFB1-PG to generate pFB1-PG-Ac66. The promoter (28 bp upstream of the *exon0* ATG start codon) and ORF sequences of *exon0* were synthesized, with Flag-encoding sequence tagged at the N-terminus of EXON0 ORF. The synthesized fragment was assembled to pFB1-PG to generate pFB1-PG-EXON0. The two plasmids were transformed individually into chemically competent DH10Bac cells to generate Ac66Flag- or EXON0Flag-expressing viruses via Tn7-mediated transposition, and the viruses were designated vAcWT^Ac66Flag^ and vAcWT^EXON0Flag^, respectively. All of the recombinants were verified by PCR and sequencing.

### Construction of recombinant plasmids

Two recombinant plasmids, namely pUC18-Ac132HA and pUC18-ME53Myc, were constructed for use in this study. The construction process involved the following steps. First, DNA sequences containing the ORF of *ac132* or *me53* and their corresponding promoters were amplified from bMON1427 using the primer pairs ac132F/ac132R and me53F/me53R (Table 4), respectively. The amplified sequences were tagged with the HA or Myc encoding sequences at the C-terminus. Subsequently, the PCR products were digested with *Sac* I and *Bam*H I and ligated to pUC18-SV40 to generate pUC18-Ac132HA and pUC18-ME53Myc. All of the recombinants were verified by PCR and sequencing.

### Virus growth curve analysis and plaque assays

To assess the growth curves of recombinant viruses, Sf9 cells were transfected with 1 µg of bacmid DNA of vAcWT, vAc51KO, vAc51ΔDnaJ, vAc51ΔRRM, or vAc51ΔCC, and the viral supernatant was collected at designated time points. Viral titer in the supernatant was determined using the TCID_50_ endpoint dilution assay (44), and viral growth curves were plotted. Plaque assays were performed as previously described (44). Specifically, Sf9 cells in a monolayer were transfected with 0.2 g bacmid DNA of vAcWT, vAc51CHA, vAc51ΔDnaJ, vAc51ΔRRM, or vAc51ΔCC. After 5 hours, a 1:1 mixture of culture medium and 4% low-melting agarose was added. At 120 h p.t., the viral plaques were photographed.

### Western blot analysis

For detection of truncated Ac51 in the cells, infected Sf9 cells were collected at designated time points and centrifuged at 6,000 rpm for 5 min. The cell pellet was suspended with PBS, mixed with loading buffer, and then heated at 100°C for 10 min. For the detection of truncated Ac51 on BVs, purified BVs were mixed with loading buffer and also heated at 100°C for 10 min. After centrifugation at 12,000 rpm for 5 min, the supernatant was resolved by SDS-10% PAGE and transferred onto a polyvinylidene difluoride (PVDF) membrane (Millipore). Western blot analysis was performed using an antibody against Ac51 (1:1,000), a rabbit polyclonal anti-HA antibody (1:2,000, 3472S, CST), or a mouse monoclonal anti-GP64 antibody (1:2,000, eBioscience) as the primary antibodies and horseradish peroxidase (HRP)-conjugated donkey anti-rabbit antibody (1:4,000; 7074S, CST) or HRP-conjugated donkey anti-mouse antibody (1:4,000; 7076S, CST) as the secondary antibodies. The signals were detected using an electrochemical luminescence system (Alliance Q9 Micro Light) according to the manufacturer’s instructions.

For the co-immunoprecipitation assay, the immunoprecipitated samples were mixed with loading buffer and then heated at 100°C for 10 min. After centrifugation, the supernatant was resolved by SDS-10% PAGE and transferred onto a PVDF membrane. Western blot analysis was performed using an antibody against Ac51 (1:1,000), a mouse monoclonal anti-Flag antibody (1:2,000, 8146S, CST), or an anti-HA antibody (1:2,000) as the primary antibodies. The HRP-conjugated donkey anti-rabbit antibody (1:4,000, A25022, Abbkine) or HRP-conjugated donkey anti-mouse antibody (1:4,000; A25012, Abbkine) was used as the secondary antibodies, which could not recognize the heavy chain of antibodies. For Ac132 detection, the HRP-conjugated donkey anti-mouse antibody (1:4,000; 18–8816-31, Rockland) was used as the secondary antibody. Therefore, these secondary antibodies eliminate interference from the heavy or light chain in western blot analysis.

### Co-IP assay

For analyzing the interaction between Ac51 and Ac66/EXON0, Sf9 cells (1 × 10^7^) were infected with vAcWT^Ac66Flag^ or vAcWT^EXON0Flag^ at a multiplicity of infection (MOI) of 5 TCID_50_/mL and collected at 48 h p.i. For analyzing the interaction between truncated Ac51 and Ac66, Sf9 cells (1 × 10^7^) were co-transfected with 10 µg bacmid DNA of vAc51ΔDnaJ, vAc51ΔRRM, or vAc51ΔCC along with 15 µg ultrapure pUC18-Ac66Flag and collected at 72 h p.t. The cells were lysed using lysis buffer (P0013, Beyotime) at 4°C with rotation for 30 min. Following centrifugation, a portion of the supernatant was saved as the input, while the remaining supernatant was pre-treated with IgG-conjugated magnetic beads (P2171, Beyotime) for 30 min. After that, the supernatant was divided into two parts. One part was incubated with 15 µL of IgG-conjugated magnetic beads, and the other part was incubated with 15 µL of Flag-conjugated magnetic beads (P2115, Beyotime) with rotation at 4°C overnight. For reverse co-immunoprecipitation experiments, the supernatant was preincubated with protein A/G agarose beads (A10001S, Abmart) for 30 min, followed by incubation with either anti-Ac51 or anti-IgG antibody with rotation at 4°C overnight. A total of 20 µL protein A/G agarose beads were then added to the mixture and incubated for 2 hours at 4°C. All beads were washed once with lysis buffer and three times with TBST (containing 0.1% Tween 20), with each wash lasting for 10 min. The immunoprecipitated samples were then subjected to western blot analysis.

For analyzing the interaction between ME53 and Ac51/Ac66, Sf9 cells (1 × 10^7^) were transfected with 15 µg pIB-ME53GFP (a plasmid provided by Professor Yang Kai’s lab) and then infected with vAcWT or vAcWT^Ac66Flag^ at an MOI of 5 TCID_50_/mL at 24 h p.t. Cells were collected at 48 h p.i. For analyzing the interaction between Ac132 and Ac51/Ac66, Sf9 cells (1 × 10^7^) were infected with vAc132HA or vAcWT^Ac66Flag^ at an MOI of 5 TCID_50_/mL and collected at 48 h p.i. The collected cells were subjected to the Co-IP assay with the anti-Flag antibody or the anti-Ac51 antibody with the same protocol described above. ME53 was detected with an anti-GFP antibody (1:2,000, TT0005S, Abmart).

### Molecular docking analysis

According to the protein ID numbers, including Ac51 (NP_054080.1), Ac66 (NP_054096.1), ME53 (NP_054169.1), and Ac132 (NP_054162.1), the corresponding sequences were retrieved from the NCBI database, and the 3D structures of the proteins were modeled using AlphaFold2 (45) software to obtain structural models suitable for subsequent molecular docking. To study the binding regions and interaction patterns between the proteins, the professional protein-protein and protein-DNA/RNA docking program HDOCK (46) was utilized for the docking analysis. The docking poses with the best docking scores were selected as the standard results for further interaction analysis. The docking scores were calculated based on the ITScorePP or ITScorePR iterative scoring functions. The more negative the docking score, the stronger the potential binding and interaction between the two molecules. Considering that the docking scores of protein-protein/RNA/DNA complexes in the PDB are typically around −200 or better, we defined a confidence score based on the docking score to represent the likelihood of binding between the two molecules, as shown below:

Confidence score = 1.0/{1.0 + e^[0.02 × (Docking_Score + 150)]^}

Specifically, when the confidence score is higher than 0.7, the two molecules are highly likely to bind; when the confidence score is between 0.5 and 0.7, the two molecules may bind; and when the confidence score is lower than 0.5, the two molecules are unlikely to bind. Finally, the binding regions and interaction patterns between the proteins were analyzed using PyMOL (http://www.pymol.org/pymol).

### Immunoprecipitation-mass spectrometry

To screen for proteins that interact with Ac51 or Ac66, Sf9 cells (1 × 10^7^) were infected with vAcWT or vAcWT^Ac66Flag^ at MOI of 5 TCID_50_/mL and collected at 48 h p.i. The cells were subjected to the Co-IP assay as described above using an anti-Ac51 antibody, anti-Flag antibody, or corresponding anti-IgG antibody as controls. The immunoprecipitated samples were subjected to SDS-PAGE, allowing all proteins to enter the stacking gel but not the separating gel, concentrating all proteins within a 1 cm range in the stacking gel. The gel containing the protein was excised and subjected to liquid chromatography-tandem mass spectrometry (conducted by the Wininnovate Bio company). By comparing the data between the control group and the experimental group, proteins that specifically interact with Ac51 or Ac66 were analyzed and identified.

### Immunofluorescence assay

To analyze the subcellular localization of proteins, uninfected or infected Sf9 cells were fixed with 4% paraformaldehyde for 10 min, followed by treatment with 0.2% Triton X-100 for 10 min. Then, the cells were incubated with 4% bovine serum albumin‌ for 30 min. Subsequently, the primary antibodies against specific proteins were added and incubated overnight. On the following day, the cells were washed twice with PBS for 10 min each. Then, the cells were incubated with Alexa Fluor-conjugated secondary antibodies (1:400, A32728/A32728, Invitrogen) for 1 hour at room temperature. After that, cells were stained with Hoechst 33342 for 10 min to indicate the cell nucleus, followed by washes with PBS three times. Finally, the cells were observed using a Leica SP8 Laser Confocal Microscope. Antibodies used in the immunofluorescence assay were as follows: anti-Ac51 antibody (1:200), anti-HA antibody (1:200), anti-Flag antibody (1:200), rabbit polyclonal anti-Myc antibody (1:200, 2278S, CST), and mouse monoclonal anti-Myc antibody (1:200, AM926, Beyotime).

### Virion purification

Sf9 cells were infected with indicated viruses, and cell supernatant was collected at 96 h p.t. BVs in the supernatant were purified as previously described (16, 47). Specifically, the virions were precipitated from the supernatant by ultracentrifugation at 4°C, followed by sucrose density gradient centrifugation to further purify the virions. Virion concentration was determined by a bicinchoninic acid (BCA) protein assay kit (20200ES76, YEASEN). The purified BV particles were then subjected to western blot analysis.

### Statistical analysis

All statistical data represents at least three independent biological replicates and was analyzed using GraphPad software, employing *t* tests for significance analysis. The fluorescence intensity of proteins and the nuclear to whole cell fluorescence ratio of proteins were measured by Image J software (48). Colocalization was analyzed using the colocalization module in Fiji software and was quantified using the Pearson correlation coefficient.

## Data Availability

All research data associated with the study are included in the article and its supplemental material.

## SUPPLEMENTAL MATERIAL

Supplemental figures jvi.01969-24-s0001.docx Figures S1 to S3.

ASM does not own the copyrights to Supplemental Material that may be linked to, or accessed through, an article. The authors have granted ASM a non-exclusive, world-wide license to publish the Supplemental Material files. Please contact the corresponding author directly for reuse.
