# Supplementary material for: Autographa californica multiple nucleopolyhedrovirus Ac51 interacts with Ac66 and facilitates its nuclear localization to promote the nuclear egress of nucleocapsids
Source: J Virol. 2025 May 28;99(6):e01969-24. doi: 10.1128/jvi.01969-24 (PMC12172431; doi:10.1128/jvi.01969-24)
Supplement: Supplemental figures — Figures S1 to S3. [file jvi.01969-24-s0001.docx]

**Autographa californica multiple nucleopolyhedrovirus Ac51 interacts with Ac66 and facilitates its nuclear localization to promote the nuclear egress of nucleocapsids**

Jianxiang Qiu^a,b^, Guo-Sheng Zhu^a^, Jiaxin Liu^a^, Longkuan Feng^a^, Zefen Pang^a^, Dandong Zhu^a^, Kanghong Chen^a^, Xiukui Yan^a^, Ao Li^c^, Chuming You^a#^, Zhixin Fang^a,b#^

^a^Biosafety laboratory, The Affiliated Guangdong Second Provincial General Hospital of Jinan University. Guangzhou 510317, Guangdong, China.

^b^GuangDong Engineering Technology Research Center of Emergency Medicine, The Affiliated Guangdong Second Provincial General Hospital of Jinan University, Guangzhou 510317, Guangdong, China.

^c^School of Life Sciences and Medicine, Shandong University·of·Technology,·Zibo 255049, Shandong, China.

**
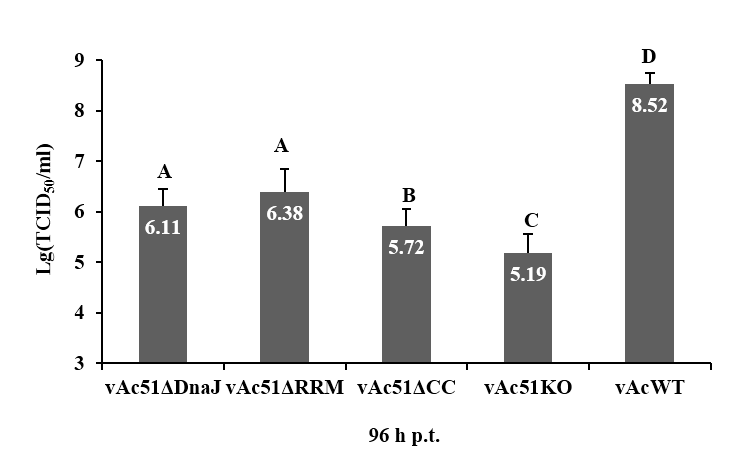
**

**Figure S1. Virus titers of recombinant viruses.** Sf9 cells, cultured in Grace’s insect cell culture medium supplemented with 10% fetal bovine serum, penicillin (100 g/ml), and streptomycin (30 g/ml), were transfected with 1 μg of bacmid DNA of indicated recombinant viruses, and cell supernatant was collected at 96 h p.t.. Virus titer was determined by the TCID_50_ endpoint dilution assay. The titer of each virus was indicated by the log value, and statistically significant differences between any two viruses were denoted using different letters.


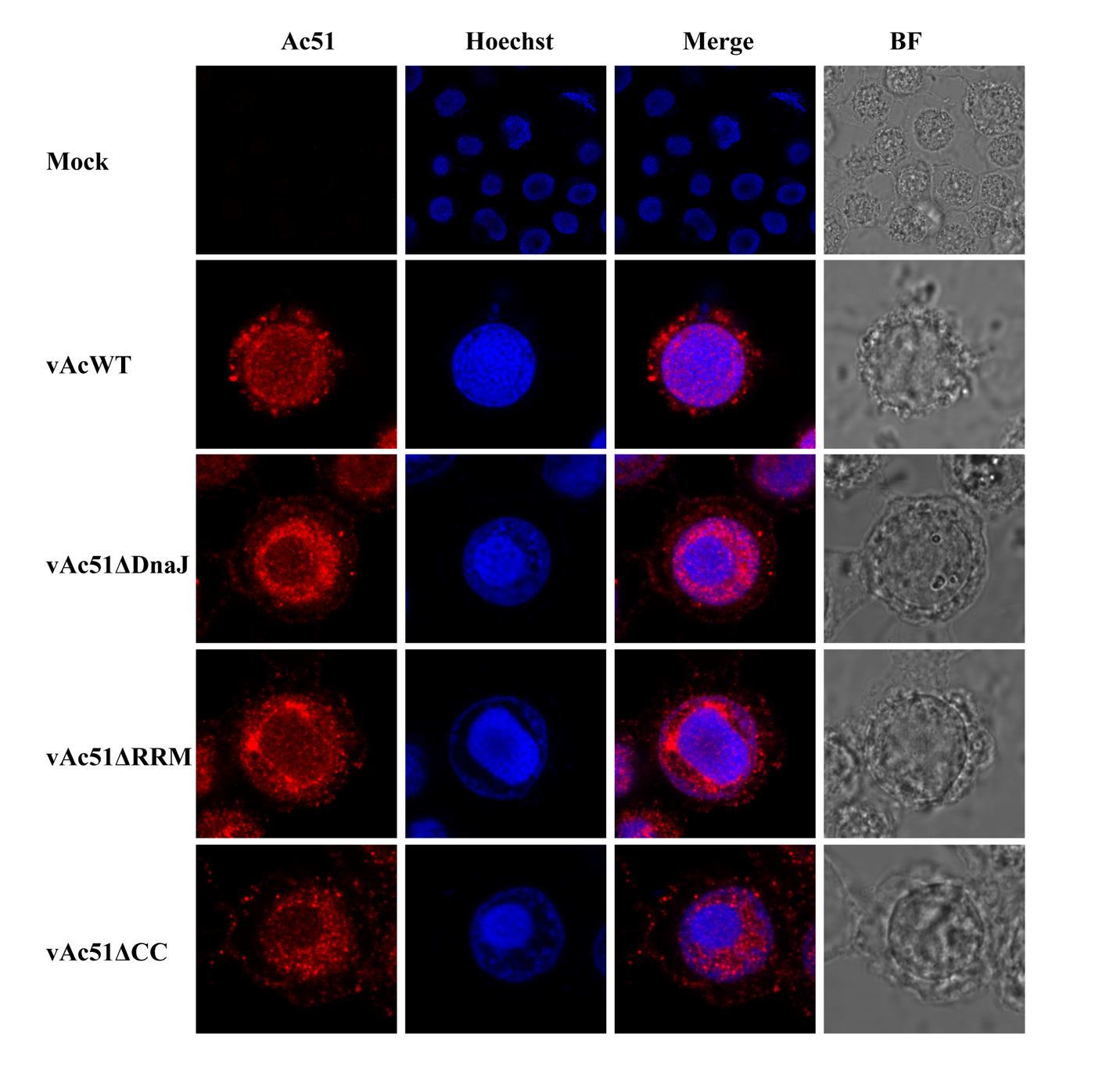


**Figure S2. Subcellular localization of truncated Ac51.** Sf9 cells were transfected with 1 μg of bacmid DNA of vAcWT, vAc51ΔDnaJ, vAc51ΔRRM, or vAc51ΔCC, and subjected to a immunofluorescence assay at 72 h p.t.. The cells were probed with the anti-Ac51 antibody, followed by a secondary antibody conjugated with Alexa Fluor™ Plus 555. Hoechst 33342 was used for nuclear staining. BF images were also captured.


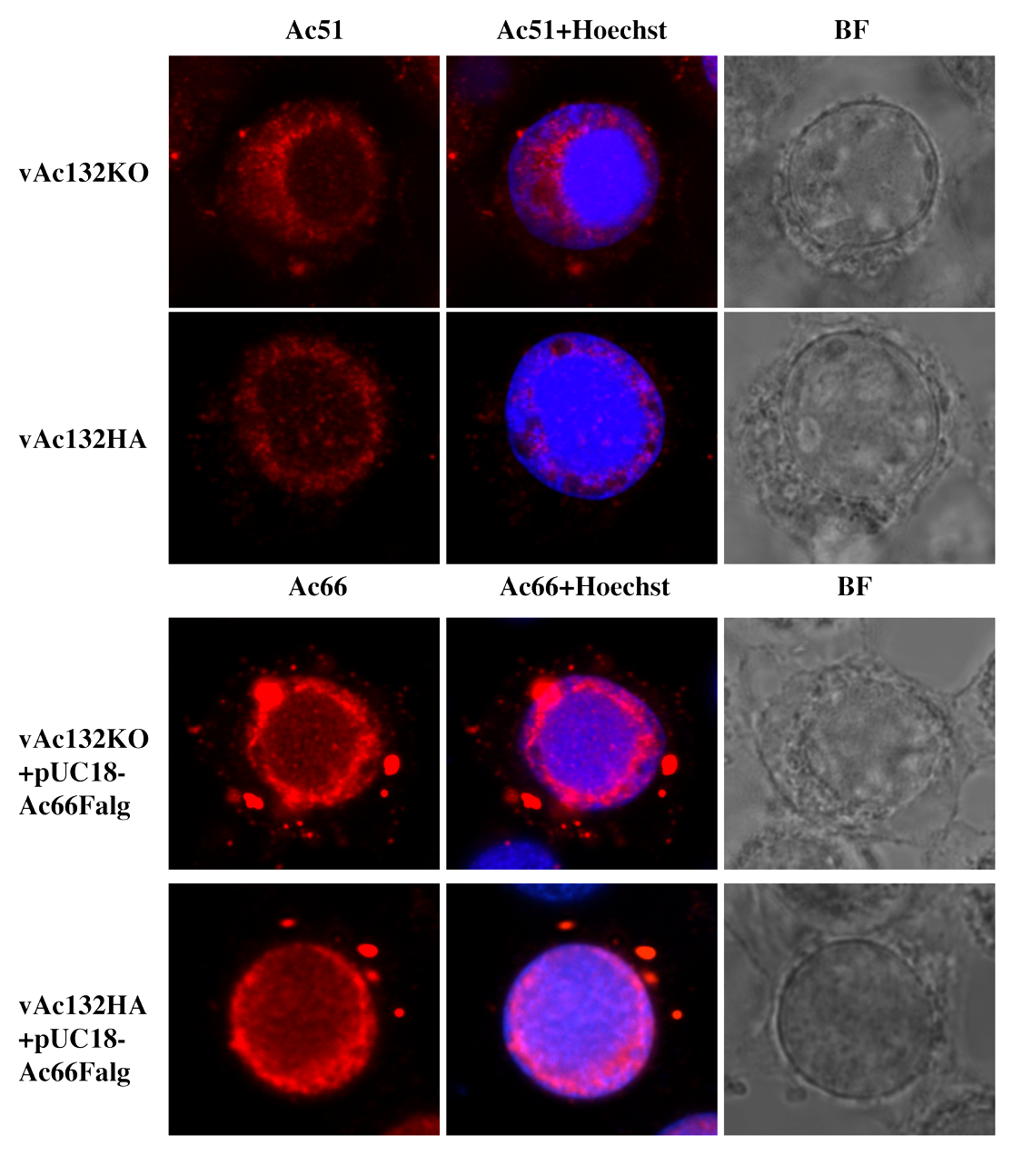


**Figure S3. Effect of *ac132* deletion on the subcellular localization of Ac51 and Ac66.** Sf9 cells were transfected with bacmid DNA of vAc132KO or an *ac132* repair virus (vAc132HA), or co-transfected with bacmid DNA of vAc132KO/vAc132HA and pUC18-Ac66Flag and subjected to a immunofluorescence assay at 72 h p.t.. The cells were probed with the anti-Ac51 antibody or anti-Flag antibody, followed by second antibodies and Hoechst 33342. BF images were also captured.
